# Supplementary material for: Developing a competency assessment framework for pharmacists in primary health care settings in India
Source: PLoS One. 2025 Mar 10;20(3):e0316646. doi: 10.1371/journal.pone.0316646 (PMC11892806; doi:10.1371/journal.pone.0316646)
Supplement: S1 File — (PDF) [file pone.0316646.s001.pdf]

Supporting file-1 Literature based Competencies and its behaviours list

Pharmaceutical Health	
Competencies	Behaviours
Health Promotion	Assess the primary healthcare needs (taking into account the cultural and social setting of the patient)<br><br>Advise on health promotion, disease prevention and control, and healthy lifestyle<br><br>Provide training programs for community staff and leaders on knowledge and skills essentials for promoting health
Medicines information and advice	Counsel population on the safe and rational use of medicines and devices (including the selection, use, contraindications, storage, and side effects of non-prescription and prescription medicines)<br><br>Identifies and utilises evidenced based sources of information on health<br><br>Provides accurate, quality and safe information and advice to patients, the public and other healthcare professionals regarding medicines<br><br>Advises patients, the public and other healthcare professionals on the safe and rational use of medicines and devices including the use, contraindications, storage, and side effects of non-prescription and prescription medicines<br><br>Identifies and utilises appropriate evidenced based sources of information on medicines<br><br>Provides medicines information in response to queries in a manner appropriate to the recipient<br><br>Manages information appropriately including documentation<br><br>Accesses reliable information to ensure cost effective use of medicines<br><br>Provides education to patients, the public, students and other healthcare professionals on medicines<br><br>Respond to Drug Information (DI) requests<br><br>Identify of Drug Related Problems (DRPs) and perform interventions
**Pharmaceutical Care**	

Assessment of medicines	Appropriately select medicines (e.g. according to the patient, hospital, government policy, etc)<br><br>Develop criteria for selecting medicines into a hospital formulary concerning pharmaceutical chemistry, formulation, quality standards and therapeutic properties<br><br>Identifies, prioritises and resolves medicines management problems<br><br>Monitors medicines use and patient adherence, as appropriate, to ensure positive clinical outcomes are achieved and patient needs are met<br><br>Encourages and facilitates patient adherence<br><br>Liaises with and provides advice to the prescriber or other healthcare professionals to ensure optimal use of medicines by patients<br><br>Contributes to the cost-effective use of medicines<br><br>Recognises and manages adverse drug reactions<br><br>Recognises and manages inappropriate medicines including dosage problems<br><br>Recognises and advises on any additional patient monitoring required<br><br>Contributes to strategies on medication management including monitoring and improving medicines use
Compounding medicines	Prepare pharmaceutical medicines (e.g. extemporaneous, cytotoxic medicines), determine the requirements for preparation (calculations, appropriate formulation, procedures, raw materials, equipment etc.)<br><br>Compound under the good manufacturing practice for pharmaceutical (GMP) medicine<br><br>Apply risk management strategies<br><br>Develop protocols for ensuring quality of prepared medicines<br><br>Develop master compounding formulas.<br><br>Optimise packaging and supplementary labelling
Dispensing	Accurately dispense medicines for prescribed and/or minor ailments and monitor the dispense (re-checking the medicines)<br><br>Perform pharmaceutical, compounding and patient-specific calculations, including pharmacokinetic and other therapeutic calculations.<br><br>Screens and manages the prescription or other medicines requisition for any

	potential clinical problems including therapeutic duplication, interaction with other medicinal products (including interactions with non-prescription<br>medicinal products, herbal products or foods),<br>incorrect dosage or duration of treatment, allergic reactions and clinical abuse and/or misuse<br><br>Consults the available patient medical and medicine history or record<br><br>Accurately report defective or substandard medicines to the appropriate authorities<br><br>Appropriately validate prescriptions, ensuring that prescriptions are correctly interpreted and legal<br><br>Document and act upon dispensing errors<br><br>Implement and maintain a dispensing error reporting system and a 'near misses' reporting system<br><br>Label the medicines (with the required and appropriate information)<br><br>Learn from and act upon previous 'near misses' and 'dispensing errors'
Medicines	Check the product label<br><br>Advise patients on proper storage conditions of the medicines and ensure that medicines are stored appropriately (e.g., humidity, temperature, expiry date, etc.)<br><br>Appropriately select medicines formulation and concentration for minor ailments (e.g., diarrhoea, constipation, cough, hay fever, insect bites, etc.)<br><br>Ensure dispensing of appropriate medicines with clear instruction for route, time, dose<br><br>Package medicines to optimise safety (ensuring appropriate re-packaging and labelling)
Monitor Medicines Therapy	Apply guidelines, medicines formulary system, protocols and treatment pathways<br><br>Ensure therapeutic medicines monitoring, impact and outcomes (including objective and subjective measures)<br><br>Identify, prioritise and resolve medicines management problems (including errors)<br><br>Support Quality Use of Medicines using review trends in medicine use & evidence-based medicine use promotion
Patient consultation and diagnosis	Structured consultations against a protocol and uses the patient consultation area appropriately

	Assess the patient's health status and concerns.<br><br>Determine the patient's actual and potential drug therapy problems<br><br>Apply first aid and act upon arranging follow-up care<br><br>Appropriately refer<br><br>Assess and diagnose based on objective and subjective measures<br><br>Discuss and agree with the patients the appropriate use of medicines, taking into account patients' preferences<br><br>Develop the patient's care plan, in partnership with the patient and in collaboration with other health professionals<br>Administer drugs to the patient using the necessary technical skills and applying the appropriate clinical Knowledge.<br><br>Document any intervention (e.g., document allergies, medicines, and food, in patient medicines history)<br><br>Obtain, reconcile, review, maintain and update relevant patient medication and diseases history
**Organization and Management**	
Budget and reimburseme nt	Acknowledge the organisational structure<br><br>Effectively set and apply budgets<br><br>Ensure appropriate claim for the reimbursement<br><br>Ensure financial transparency<br><br>Ensure proper reference sources for service reimbursement
Human Resources management	Identity and manage human resources and staffing issues<br><br>Participate, collaborate, advise in therapeutic decision-making, and use appropriate referral in a multi-disciplinary team<br><br>Recognise and manage the potential of each member of the staff and utilise systems for performance management (e.g., carry out staff appraisals)

	Recognise the value of the pharmacy team and of a multidisciplinary team<br>Support and facilitate staff training and continuing professional development.
Improvement of service	Identify and resolve risk management issues to improve quality and safety of medicine use and services<br><br>Initiate and implement programs or interventions to improve dicine use systems<br><br>Recognises quality as a core principle of medicines management and healthcare provision<br><br>Understands the role of policies and procedures in the organisational structure and in the provision of healthcare<br><br>Contributes to regular audit activities and reports and acts upon findings<br><br>Identifies and evaluates the evidence-base to improve the use of medicines and services, including risk management<br><br>Uses feedback from complaints and audit to improve and develop services in conjunction with their manager<br><br>Implements a system for documentation and record keeping
Procurement	Access reliable information and ensure the most cost-effective medicines in the right quantities with the appropriate quality<br>Prepare indents for procurement including list of drugs indexed on------ in consultation with other members of the team<br><br>Develop and implement contingency plan for shortages<br><br>Efficiently link procurement to formulary, to push/pull system (supply chain management) and payment mechanisms<br><br>Ensure there is no conflict of interest<br><br>Select reliable supplier of high-quality products (including appropriate selection process, cost effectiveness, timely delivery)

	Supervise procurement activities<br><br>Understand the tendering methods and evaluation of tender bids<br><br>Implement and supervise procurement systems
Supply chain and management	Demonstrate Knowledge in store medicines to minimise errors and maximise accuracy<br><br>Ensure accurate verification of rolling stocks<br><br>Ensure effective stock management and running of service with the dispensary<br><br>Ensure logistics of delivery and storage<br><br>Implement a system for documentation and record keeping<br><br>Take responsibility for quantification of forecasting<br><br>Develop efficient inventory system management<br><br>Develop procedures for medicine storage and delivery<br><br>Demonstrates Knowledge of the medicines supply chain and selects reliable and appropriately authorised suppliers of medicinal products<br><br>Ensures that medicines are stored in appropriate facilities and environmental conditions to ensure stability, quality, and safety of the medicinal product over its shelf life<br><br>Stores medicines in a safe, organised, systematic and secure

	manner<br><br>Works with documented policies and procedures to implement an effective stock management and rotation system, including systems for forecasting patient needs and demands and contingency plans for shortages and discontinuations<br><br>Works with documented policies and procedures to manage the recall of medicines, including the assessment of impact on patient care and required patient follow-up<br><br>Ensures there is no conflict of interest or inappropriate inducements in the sourcing and supply of medicines<br><br>Demonstrates an understanding of the legislative framework and requirements that govern the distribution of medicinal products including Good Distribution Practice (GDP)<br><br>Encourages patients to return any unused, unwanted, or expired medicines to the Pharmacy for safe disposal
Workplace management	Create and maintain collaborative professional relationships.<br><br>Participate in the delivery of collaborative health services.<br><br>Address and manage day to day management issues<br><br>Demonstrate the ability to take accurate and timely decisions and make appropriate judgments<br><br>Ensure the production schedules are appropriately planned and managed<br><br>Ensure the work time is appropriately planned and managed<br><br>Ensures effective handover between team members or to another healthcare professional to ensure continuity of patient care<br><br>Participates, collaborates, and advises on therapeutic decision-making and uses appropriate referral in a multi professional team<br><br>Works effectively with the documented procedures and policies within the

	workplace<br><br>Understands their role in the organisational structure and works effectively within the management structure of the organisation<br><br>Works effectively with their supervising and superintendent Pharmacist (s)<br><br>Identifies pharmacy resource requirements and manages those resources effectively as appropriate to their level of responsibility<br><br>Establishes commitment from team members through good relationships, models the kind of behavior expected to benefit good pharmaceutical patient care
**Professional / Personal**	
Communication skills	Communicate clearly, precisely, and appropriately while being a mentor or tutor<br><br>Uses effective verbal, non-verbal, listening, and written communication skills to communicate clearly, precisely and appropriately<br><br>Communicate effectively with health and social care staff, support staff, patients, carer, family relatives and clients/customers, using lay terms and checking understanding<br><br>Demonstrate cultural awareness and sensitivity<br><br>Tailor communications to patient needs<br><br>Use appropriate communication skills to build, report and engage with patients, health and social care staff and voluntary services (e.g., verbal, and non-verbal)<br><br>Demonstrates influencing and negotiation skills to resolve conflicts and problems<br><br>Deliver an education session to an individual or group.<br><br>Listens to patients and respects their views about their health and medicines<br><br>Apply interpersonal communication skills to address problems.<br><br>1.Analyse the problem or issue to be addressed and the possible solutions<br>2. Engage with others as appropriate to resolve the identified problem
Continuing Professional Development (CPD)	Understands and accepts the importance of life-long learning for pharmacists<br><br>Document CPD activities<br><br>Engage with students/interns/residents<br><br>Evaluate currency of Knowledge and skills<br><br>Evaluate learning

	Identify if expertise needed outside the scope of Knowledge<br><br>Identify learning needs<br><br>Recognise own limitations and act upon them<br><br>Reflect on performance
Legal and regulatory practice	Apply and understand regulatory affairs and the key aspects of pharmaceutical registration and legislation<br><br>Apply Knowledge in relation to the principles of business economics and intellectual property rights including the basics of patent interpretation<br><br>Comply with legislation for drugs with the potential for abuse<br><br>Engage with health and medicines policies<br><br>Understand the steps needed to bring a medicinal product to the market including the safety, quality, efficacy and pharmacoeconomic assessments of the product<br><br>Demonstrate Knowledge in legal and regulatory practices along pharmaceutical supply chain<br><br>Demonstrate Knowledge in healthcare systems regarding policies, financing, information systems, workforces, service delivery, and access to essential medicines and health technologies<br><br>Practise within legal requirements.<br><br>Manage actual and potential illegal, unethical, or unprofessional actions or situations in practice.<br><br>Document activities of practice in compliance with federal and provincial/territorial legislation, standards, and policies.<br><br>Apply federal and provincial/territorial workplace, occupational health and safety, and other related legislation to the practice setting.<br><br>Demonstrates an awareness of other legislation relevant to their practice setting including as appropriate data protection law, health and safety law, employment law, consumer law, equality law and intellectual property rights
Professional and ethical practice	Demonstrate awareness of local/national codes of ethics<br><br>Ensure confidentiality (with the patient and other healthcare professionals)<br><br>Obtain patient consent (it can be implicit on occasion)<br><br>Take responsibility for own action and for patient care

	Apply ethical principles in the decision-making process.<br><br>Manage situations of actual and perceived conflict of interest.<br><br>Select appropriate methods to share documentation within the circle of care and facilitate patient care.
Quality Assurance and Research in the workplace	Apply research findings and understand the benefit risk (e.g., pre-clinical, clinical trials, experimental clinical-pharmacological research, and risk management)<br><br>Audit quality of service (ensure that they meet local and national standards and specifications)<br><br>Develop and implement Standing Operating Procedures (SOP's)<br><br>Ensure appropriate quality control tests are performed and managed appropriately<br><br>Ensures medicines are not counterfeit and quality standards<br><br>Identify and evaluate evidence-base to improve the use of medicines and services<br><br>Identify, investigate, conduct, supervise and support research at the workplace (enquiry-driven practice)<br><br>Implement, conduct, and maintain a reporting system of pharmacovigilance (e.g., report Adverse Drug Reactions)<br><br>Handle hazardous products safely by minimizing personal exposure and reducing environmental contamination<br><br>Initiate and implement audit and research activities<br><br>Apply Knowledge, research skills and professional judgment to the decision-making process<br><br>Critically analyse and develop solutions to problems in pharmacy practice.<br><br>Applies principles of scientific enquiry to investigate a<br>medicine or practice related issue<br><br>Make decisions using an evidence-informed approach.
Self-management	Apply assertiveness skills (inspire confidence)<br><br>Ensure punctuality<br><br>Prioritise work and implement innovative ideas<br><br>Collaborate with others to work towards the target, take responsibility and respect diversity

	Demonstrate caring and service mind<br><br>Demonstrate flexibility and adaptability in uncertain situations<br><br>Demonstrate systems thinking in understanding complex situations and interconnections between things & Continuous self-improvement<br><br>Respect the personal characteristics, rights, preferences, values, beliefs, needs and cultural and linguistic diversity of patients and other clients, including Aboriginal and Torres Strait Islander peoples<br><br>Consider the impact of the physical environment
