# Supplementary material for: Developing a competency assessment framework for pharmacists in primary health care settings in India
Source: PLoS One. 2025 Mar 10;20(3):e0316646. doi: 10.1371/journal.pone.0316646 (PMC11892806; doi:10.1371/journal.pone.0316646)
Supplement: S2 File — (PDF) [file pone.0316646.s002.pdf]

Supporting File-2 Pharmacists Role based Competencies and behaviours List

Role	S.N.	Competencies	S.N.	Behaviours
Pharmacy Manager	**1.**	Knowledgeable about the layout, Infrastructure of the Pharmacy and how to adapt it to optimise pharmacy services including inventory management	1.	Assessment of the existing infrastructure and identifying gaps and opportunities for improvement.
			2.	Initiation and supervision of the needed infrastructure modification
			3.	Implement suitable inventory techniques
			4.	Appropriate labelling of places of segregation of Materials, segregation of the various areas in the Pharmacy & Pharmacy Store including the place for quarantine.
	2.	Knowledgeable about drugs, consumables, and materials to be kept in store, their nature, use, and storage conditions required	5.	Prepare list of articles to be stored, and their storage requirements
			6.	Verify that infrastructure is in place to ensure that all the required conditions of storage are met.
			7.	Ensure premises and products are kept free from pests
			8.	Facilitate the appropriate pest control measures in place, and document them
	**3.**	Be able to maintain and ensure the required storage conditions	9.	Arrange and organize the medical products
			10.	Verify that the articles are maintained according to the required storage condition
			11.	Regular checking of storage conditions and physical inspection of medical products
			12.	Timely corrective measures
			13.	Label the various storage shelves/racks/places/pharmaceuticals (with the required and appropriate information)

			14.	Stores medicines in a safe, organised, systematic and secure manner
	**4.**	Knowledgeable about the procedure for all types of planning and procurement (Pool Procurement)	15.	Based on epidemiological condition identify the drugs that would be needed by the patients in the PHC and subcentres.
			16.	Based on the population, past consumption pattern, prescribing practice of the clinicians and the seasonality of disease incidence, project the quantities required and the time frame of procurement.
			17.	Prepare the indent in the required format and get it approved by the medical officer in charge.
			18.	Justify and defend the requirement mentioned in the indenting form and if required, it may be amended
			19.	Ensuring compliance to local purchasing rules and adequate documentation
			20.	Prepare indents in consultation with healthcare providing team members
			21.	Develop and implement contingency plan for shortages/ stock out
			22.	Ensure there is no conflict of interest
	**5.**	Able to manage the inventory correctly	23.	Verify that the drugs supplied correspond to the description and quantity as required.
			24.	Monitor the consumption and ensure that it follows the projection based on which the indent was done
			25.	Be aware when the reorder point is reached
			26.	Track the delivery status of ordered supplies and alert the medical officer if required
			27.	Initiate alternate procurement procedures if needed to prevent a stockout (e.g.: Local purchase,
			28.	Request medical officer to alter the prescription if needed.
			29.	Inventory analysis and interpretation -ABC/VED
			30.	Works with documented policies and procedures to implement an effective stock management

			31.	Prepare the list of habit forming (example- Tramadol HCl) and develop systems to track them
			32.	Prepare the list of slow-moving medical product and develop systems to track them
			33.	Where the use of the slow-moving drugs appears unlikely or short expiry , transfer them to institutions which need them, without delay and document as per the guideline.
	**6.**	Able to perform and fulfil the duties as a member of different committees in the institution or representing it.	34.	Participating in the committee meetings like Pharmacy (drug) and therapeutic committee, Annual Indent committee, Purchase committee, Condemnation committee and Prescription audit committee and provide inputs wherever required
			35.	If required, duly inform the concerned persons about the committee meeting
			36.	If required, documentation of the meetings participated.
			37.	Dissemination of the minutes of the meeting to the concerned persons.
			38.	Identify the risk situations and list down the risk management plan
	**7.**	Able to manage Drugs and vaccine Distribution Management system (DVDMS)/ eNiramaya, and others existing IT system (Adequate digital literacy)	39.	Knowledgeable about the government orders /circulars on Drugs and vaccines distribution management system
			40.	Attend training /workshops on Drugs and vaccine Distribution Management system (DVDMS)
			41.	Use the Drugs and vaccine Distribution Management system (DVDMS) optimally,
			42.	Within the specified period, all Input data should be updated on DVDMS platform
			43.	Circulate monthly DVDMS report to all concerned and track the follow up
			44.	Clarify other members doubt about the IT based Pharmacy Management system and support them to
			45.	Knowledge on use of computers including MS Office and use of internet for professional use for daily office work
	**8.**	Able to conduct institutional store verification, condemnation and maintenance of all registers and records relevant to Pharmacy and pharmacy store	46.	Conduct periodical pharmacy Stock internal Verification
			47.	Condemnation of un-serviceable medical products
			48.	Maintenance of registers and records

	**9.**	Knowledgeable about the information and directions from authorities relating to banned and Not of Standard Quality (NSQ) medicines and acting appropriately	49.	Keep track of government notifications on frozen or NSQ medical products.
			50.	Passing information to medical officer in-charge with name and batch number for banned and NSQ medical products
			51.	If frozen items or NSQ medical products are released take necessary action to retrieve them
			52.	Segregating the NSQ drugs from the main stock
			53.	Labelling the area where NSQ drugs are segregated, as "not for use"
			54.	Recording and reporting of condemnation and disposal processes of NSQ medical products
			55.	If require, Safe disposal of NSQ medical products
**To assist the medical officer in preparation and implementation of different projects and programs**	**10.**	Able to assist the medical officer in preparation and implementation of different health projects and programs	56.	Have Knowledge about various National/ State Health Programs
			57.	Actively participate in preparation and implementation of different health projects and programs
			58.	Assist Medical Officer in management of relevant medical products (Estimating the requirement of medical products to their use)
			59.	Assist medical officer in documentation relevant to the use of medical products
Dispensing Medical Products	**11.**	Knowledgeable about drug details for dispensing including generic/brand names, usage (Indication), dosage (Quantity and frequency), route of administration, how to use and drug interaction/compatibility,	60.	Prescription validation
			61.	Dispense drugs to patient with due diligence
			62.	Identify prescription errors and potential dispensing errors
			63.	Provides the medical products conveniently and adequately ancillary labelled (Patient name Dosage, Shake well before use, For external use). Those requiring cold temp storage to be adequate packed, and patient given necessary instructions for storage
			64.	Document and act upon dispensing errors
			65.	Implementation of the reporting system for dispensing errors and near misses
			66.	Knowledgeable about the possible drug interaction
			67.	If available, refer the record for patient's medical and medicine use history
	**12.**	Able to monitor medicines use and patient adherence, as appropriate, to ensure positive clinical outcomes	68.	Monitoring the medication adherence of Chronic diseases like, Tuberculosis and diabetes etc.

	**13.**	Encourage and facilitate the patient for medication adherence	69.	Counsel the patient for the purpose of medication, storage, dosage, timing, way/method of usage/administration, drug interaction/ compatibility, adverse drug reaction, diet and lifestyle modifications
			70.	Resolve patient queries on medication
			71.	Provide written information material (Patient Information Leaflet) to assist patient in correct usage of medicines
	**14.**	Able to encourage optimal use of medical products	72.	Encourages patients to return any unused, unwanted, or expired medicines to the Pharmacy for safe disposal.
			73.	Recording and Reporting System for returned medical products
	**15.**	Able to recognise and support Primary Health Care team in managing adverse drug reactions	74.	Filling up the adverse drug reaction Monitoring Reporting form and make arrangement to send nearby Pharmacovigilance Centre (Medical College - Usually have Pharmacovigilance Centre)
**Outreach to the community**	**16.**	Able to assist in organizing community outreach camps	75.	Prepare the listof medical products required for outreach camps and arrange their logistics
			76.	Knowledge about Bio Medical Waste Management --Collection, Segregation and Transportation
	**17.**	Able to support in disaster management preparedness	77.	Be an active Member of the disaster management team
			78.	Be trained in various disaster management aspects, with special reference to medicine handling, preparing emergency medicine lists, following government guidelines on acceptance of medical products donations
			79.	Be trained and equipped in First Aid, CPR and other related aspects which could be needed in handling disaster situations
**Ensure compliance to regulations on medical products**	**18.**	Able to guide the primary care team of health facility to ensure compliance to laws and regulations as applicable to medical products	80.	Acquire and update Knowledge on all the laws and regulations that apply to medical products
			81.	Assess the current operational practices in regard to regulatory mechanism compliance in respect to antibiotic resistance, Psychotropic drugs and others.
			82.	Possible area of Improvement including the narcotics and psychotropic agents' medical products in terms of regulatory compliance
			83.	Aware about the State Drugs policy
**Professional Practice**	**19.**	Knowledgeable about Roles and Responsibilities	84.	Knowledgeable about Pharmacist's roles and responsibilities

**Ethical practice**	**20.**	Able to comply with the ethical practices	85.	Be well versed with the Code of Ethics of Pharmacy Council of India and as given in Pharmacy Practice Regulation
			86.	Ensure confidentiality of the patient's illness, and his/her treatment
			87.	Manage situations of actual and perceived conflict of interest
**Communication**	**21.**	Able to communicate effectively in normal and emergency situation	88.	Ensuring the clear, precise, and effective Communication with health and social care staff, support staff, patients, carer, family relatives, using lay terms
			89.	Demonstrate Socio-cultural awareness and sensitivity
			90.	Provides medical products information in response to queries in a manner appropriate to the recipient
			91.	Demonstrates skills to resolve the dispute
			92.	Listens to patients and respects their views about their health and medicines
			93.	Able to manage Official Communication including committee meeting-Letters/ noting etc.
			94.	Maintain inter professional communication with other primary health care team members
**Workplace Management**	**22.**	Able to manage workplace	95.	Participate as a team member in delivering health services
			96.	Ability to take appropriate and timely decisions regarding workplace management
			97.	Able to plan and manage work time appropriately
			98.	If require, able to follow the handover process with the colleagues or other team members
			99.	Understand Pharmacist roles/ responsibilities and works in harmony within the organizational structure
**Emergency Role /Clinical Role in absence of Medical Officer or other Staff**	**23.**	Able to Carry out basic tests/ Checks, to support other health professionals	100.	Carrying out Point of Care (PoC) tests like; basic tests/checks, Blood pressure, Blood Glucose level using Glucometer, Pulse Oximetry, Peak flow tests, Weight, height checks and BMI Calculations, Snellen's Chart, Pulse, Respiration Rate, Hb using strips, Pregnancy testing, other spot tests using testing kits, etc.
	**24.**	Able to handle medical devices/equipment	101.	Assemble/fit, use Oxygen cylinder, along with administration of oxygen as prescribed by the Physician
			102.	Provide Nebulization
			103.	Wound dressing/management

	**25.**	Able to manage minor ailments by recommending medicines (Odisha State Guidelines)	104.	Be able to assess patients' minor ailments, and recommend medicines, within the ambit recommended/prescribed by the Govt of Odisha for Pharmacists in primary care settings.
			105.	Be able to assess and refer the patient to the higher centres
**Continuing Professional Development**	**26.**	Willingness to participate in Continuing Professional Education/ Development programmes to update Knowledge and practice	106.	Attend online and offline training programmes to upgrade one's Knowledge, attitude and skills
			107.	Be part of the various training programmes under the NHM and various health programmes of the MoHFW & State Health department
