# Supplementary material for: Developing a competency assessment framework for pharmacists in primary health care settings in India
Source: PLoS One. 2025 Mar 10;20(3):e0316646. doi: 10.1371/journal.pone.0316646 (PMC11892806; doi:10.1371/journal.pone.0316646)
Supplement: S3 File — (PDF) [file pone.0316646.s003.pdf]

Supporting File-3 Competency Assessment Tool

Competency Assessment Tool- Pharmacists

C. Pharmacists' Domains[1]

Domains	Codes
Pharmacy/ Store Manager	1
Assistance to Medical Officer in Preparation and Implementation of different Projects and Programmes	2
Dispensing Medical Products	3
Outreach to the community	4
Ensure compliance to regulations on drugs	5
Professional Practice	6
Ethical practice	7
Communication Skill	8
Workplace Management	9
Emergency Role /Clinical Role in absence of Medical Officer	10
Continuing Professional Development	11

This tool is meant to assess the competencies of Pharmacists in terms of **Knowledge**[2], **Skills**[3] and **Attitudes**[4] through **Questionnaire, Direct Observation of Procedural skills**, **Mini Clinical Evaluation (Selected Conditions)** and **Simulation Exercise**[5]**.**

C.1. Questionnaire (Knowledge and Attitude)

S. N.	Points	Knowledge/ Attitude/ Skill	Response (Yes/No)	Remarks
1.	Do you know about the availability of any guidelines (State/ National) that defines the size of Pharmacy for Primary health Centre?	Knowledge		

1 Broadest Category of Competencies
2 Concepts and Theories
3 Use of techniques to integrate knowledge into practice
4 A person’s feelings, values, and beliefs, which influence their behaviour and the performance of tasks
5 A Simulation Exercise (SimEx) is a fabricated situation, similar to happening in real life.

2.	Do you know, what should be the size/area of Pharmacy of your Primary Health Care Centre?	Knowledge		
3.	Do you know about any of Pharmacy Infrastructure assessment tool (Format) ? / Do you have prepared any tool to assess Pharmacy Instructure for your facility?	Knowledge		
4.	Do you think, Infrastructure assessment helps in identifying gaps in your Pharmacy?	Attitude		
5.	In your sense, if the identified gaps get fulfilled in your Pharmacy, can lead to better performance of your Pharmacy?	Attitude		
6.	Do you think, the inventory management techniques could support the Pharmacist in managing the medical products better?	Attitude		
7.	Do you know about the parameters for segregation of various products in the pharmacy and pharmacy store including the quarantine place?	Knowledge		
8.	Do you think, the labelling of segregated places for medical products is a useful exercise?	Attitude		
9.	Do you know that the pest control is also essential component of Good Storage practice in Pharmacy and pharmacy store?	Knowledge		
10	Do you know that the frequent pest control is necessary for Pharmacy and pharmacy store?	Knowledge		
11	Have you ever observed any quality issues during storage of medical products in your pharmacy and Pharmacy store during your service?	Attitude		
12	Do you think that regular checking of storage conditions (Temperature) is essentially required for medical products?	Attitude		
13	Would you like to use the medical products whose appearance has been changed during the storage?	Attitude		
14	Do you think, the storage of medical products in safe and secure manner could help the Pharmacist in reducing pilferages?	Attitude		
15	Do you know about the methodology for forecasting the requirement of medical products?	Knowledge		
16	Do you feel the need of forecasting of requirements for medical products in your facility?	Attitude		
17	Do you know about the local purchasing rules for medical products?	Knowledge		
18	Do you know how to document the local purchasing of medical products?	Knowledge		
19	Do you think the documentation is important in the	Attitude		

	case of local purchasing of medical products?			
20	Do you prefer to engage healthcare providing team members of your facility before preparing the indent?	Attitude		
21	Do you feel that the contingency plan can help the Pharmacist in reducing stock out of medical products?	Attitude		
22	Do you know the concept of conflict of Interest?	Knowledge		
23	Do you prefer to give declaration of the non-conflict of interest for local purchasing of medical products?	Attitude		
24	Do you know what the right time would be to make indent (Reorder)?	Knowledge		
25	Do you know how much time usually it requires after raising indent to get medical products delivered to your facility?	Knowledge		
26	Do you prefer to consider the usual delivery time (Ordering to receiving) before making an indent for medical products?	Attitude		
27	Do you know the alternative medicines in case of stock out?	Knowledge		
28	Do you prefer to suggest medical officers to alter the prescription in case of stock out?	Attitude		
29	Do you know which habit-forming drugs are available in your facility?	Knowledge		
30	Do you know how to track the use of habit-forming medical products?	Knowledge		
31	Are you willing to take preventive measures for safeguarding habit-forming medical products?	Attitude		
32	Do you know which are slow moving medical products in your facility?	Knowledge		
33	Do you prefer to take efforts to prevent overstocking of slow-moving medical products?	Attitude		
34	Do you prefer to attend the various committee of which Pharmacist is a member like annual indent committee, purchase committee, Condemnation committee and Prescription audit committee, RKS, Purchase committee, Infection control committee, quality assurance committee, IDSP meetings?	Attitude		
35	Do you prefer to disseminate the minutes of the meeting with the concerned persons, if required?	Attitude		
36	Do you know about the circulars/government orders for I.T. based Drugs and vaccines management system?	Knowledge		

37.	Are you interested in attending training on Drugs and vaccines management system?	Attitude		
38.	In your sense, do you believe that Drugs and vaccines management system can improve Pharmacist's performance?	Attitude		
39.	Do you know how to resolve the issues/ doubts encountered by other users of DVDMS?	Knowledge		
40.	Are you willing to make SOP (If not available) for internal stock verification for your pharmacy/ store?	Attitude		
41.	Do you know the term Not of Standard Quality (NSQ) Medical Products?	Knowledge		
42.	In the case of NSQ drugs availability in your Pharmacy or store, do you prefer to segregate it from the main stock?	Attitude		
43.	Do you know how to record the condemnation and disposal processes to be followed for NSQ medical products?	Knowledge		
44.	In your sense, is it worth to record the condemnation and disposal processes followed for NSQ medical products?	Attitude		
45.	Do you know the mechanism to dispose the Not of Standard Quality (NSQ) medical products?	Knowledge		
46.	Do you know about the various National/ State Health Programs like Niramaya, RNTCP, VBDCP?	Knowledge		
47.	Do you know how to estimate the requirement of medical products for the health programs?	Knowledge		
48.	Do you prefer to make sure that the right medical product is on the way to dispensing to the patients?	Attitude		
49.	Do you know what the government guidelins/ Standard Treatment Guidelines/ Standard Treatment Workflows are required to be referred to identify the issues in prescription?	Knowledge		
50.	Do you understand the term dispensing errors?	Knowledge		
51.	Do you know what are the factors which leads to dispensing errors?	Knowledge		
52.	Do you prefer to document the encountered errors in prescription and dispensing of medical products?	Attitude		
53.	Do you prefer to fix auxiliary labelling the medical product before dispensing?	Attitude		
54.	do you prefer to report dispensing errors and near misses in your Pharmacy?	Attitude		

55.	In case of confusion, do you prefer to refer to the patient's medical and medication use history, if available?	Attitude		
56.	Do you think that the patient's queries on medication are worth to entertain?	Attitude		
57.	Do you prefer to establish a system for collection and documentation of returned medical products for safe disposal?	Attitude		
58.	Do you know how to report adverse drug reaction events?	Knowledge		
59.	Do you prefer to report the adverse drug reaction events?	Attitude		
60.	Do you know the term health promotion?	Knowledge		
61.	Do you know how to communicate the public for health promotion?	Knowledge		
62.	Do you know what IEC materials supplied by government available to inform public on disease/ illness prevention and health promotion?	Knowledge		
63.	Do you prefer to participate, as a trainer, in training community staff and leaders on disease/ illness prevention and health promotion?	Attitude		
64.	Do you know what are the sources shared by government for health information?	Knowledge		
65.	Do you prefer to advise public on safe and rational use of medical products?	Attitude		
66.	Do you think it is worth to prepare the list of medical products and arrange their logistics required for outreach camps?	Attitude		
67.	Do you know what the role of Pharmacist in the disaster management team could be?	Knowledge		
68.	Do you prefer to be the part of disaster management team?	Attitude		
69.	Do you know the availability of government guidelines for accepting medical products donations?	Knowledge		
70.	Do you know how to manage medicines during disaster situation?	Knowledge		
71.	If require, could you prefer to provide first aid during disaster management?	Attitude		
72.	Do you know what the laws/ acts are applied to medical products as supplied to health facilities?	Knowledge		
73.	Do you prefer to make an assessment of the current operational practices in regard to	Attitude		

	regulatory mechanism compliance?			
74.	Do you know what the potential areas of improvement including the narcotics and psychotropic agents medical in terms of regulatory compliance could be?	Knowledge		
75.	Do you know the term "Potential for abuse"?	Knowledge		
76.	Do you feel worth to put effort to discourage the abuse of medical products?	Attitude		
77.	Do you know about the latest state drug policy?	Knowledge		
78.	Do you think it is worth to follow the code of ethics of Pharmacy Council of India and as given in Pharmacy Practice Regulation?	Attitude		
79.	Do you think it is worth to maintain the confidentiality of patient's illness, and his/her treatment?	Attitude		
80.	Do you know what the Pharmacist's patient care responsibilities are?	Knowledge		
81.	Do you know the term "Conflict of Interest" and "Perceived conflict of Interest"?	Knowledge		
82.	Are you willing to disclose the conflict of interest?	Attitude		
83.	Do you think the good practices could be helpful in minimizing/ avoiding the conflict of Interest?	Attitude		
84.	Do you prefer to maintain professional relationship with other primary health care team members?	Attitude		
85.	Do you prefer to participate as a team member in delivering health services?	Attitude		
86.	Do you know the government of Odisha's order for dispensing medicines for specific conditions in absence of medical officer?	Knowledge		
87.	Do you know what those conditions for which government of Odisha permits to dispense medicines in absence of medical officer (without medical officer's prescription)?	Knowledge		
88.	Do you know the list of medicines for which government of Odisha has empowered Pharmacist to dispense in absence of medical officer (without medical officer's prescription)?	Knowledge		
89.	Do you know how to diagnose (Diagnostic tests/ Sign and Symptoms) the cases of Malaria, Upper Respiratory Tract Infection, Scabies?	Knowledge		
90.	Do you know how to select the medicine for a condition from permissible medicine list, for	Knowledge		

	example- selection of Antibiotic for fever- Azithromycin/ Cefixime / Amoxycillin ± Clavulanic Acid. ?			
91.	Do you know when the patients are supposed to be referred to the higher health facilities?	Knowledge		
92.	Do you prefer to refer the patient to the higher facilities, if required (In absence of medical officer)?	Attitude		
93.	Do you know what Continuing Professional Education/ Development programmes are available for pharmacists?	Knowledge		
94.	If programs are available, would you prefer to attend those Continuing Professional Education/ Development programmes?	Attitude		
95.	Do you know the governments training programs for pharmacists?	Knowledge		
96.	Do you prefer to attend government organized programs for pharmacists?	Attitude		

C.2. Observational Tools (Skills)

Score Definition

A. None—No demonstrated skills at all/does not perform the task(s) completely
B. Limited Demonstrated very limited strengths/skills in this area
C. Some—Demonstrated some ability/skills in this area.
D. Strong—Demonstrated strong skills/strength in this area.
E. Excellent—Demonstrated excellent skills/strength in this area.
F. Not applicable
G. Don't know- Not even heard about that skill
H. Skill limitation is clearly related to resource limitations

S. N.	Observation Points	Means of Verification	Response (Score)
1.	Realignment of Infrastructure	Identification of the areas of realignment<br><br>Physical verification of the feasibility of realignment	
2.	Labelling of Places of segregation in the Pharmacy and Pharmacy Store	Places labelled or not<br><br>Quality of label whether the label mark is permanent	
3.	Identification of Storage area in storeroom including shelves (Objective is to maintain potency and easy accessibility)	Whether the storage area is identified for the temperature sensitive and moisture sensitive medical products.	

4.	Organizing the Medical Products in Pharmacy and Pharmacy store	Whether the storage place is labelled with type /Name	
5.	Maintenance of Pest control record	The display of pest control record with essential detail	
6.	Storage of Medical Products in safe and secure manner	Identification of Medical Products susceptible to misuse and abuse<br><br>Medical products kept under lock and key (Specially for habit forming medical products)	
7.	Filling up the Indenting Form	Indenting Form Parameters<br><br>Strength, type and Quantity of Medical Products	
8.	Use DVDMS	Data entry -Inputs Up to date<br><br>Generate Monthly Report	
9.	Identify area of Improvement and tracking the progress based on DVDMS	Listing of the areas require improvement<br><br>Sharing those areas requiring improvement with other team members<br><br>Tracking the action taken on areas require Improvement	
10.	Condemnation Policy / Condemnation Committee functionality	Policy document<br><br>Committee Constitution document<br><br>Last Committee meeting minutes	
11.	Register maintenance -Pharmacy	Register availability for Medical Products stock<br><br>Correctness of Entries	

		Completeness of entries	
12.	Tracking of Government notifications on frozen or NSQ medical products	Availability of Records<br><br>Recent State Government notification available<br><br>Central Drug Standard Control Organization (CDSCO) notification	
13.	Identification of frozen or NSQ Medical Products	Matching the medical products with product name and batch number of Government notification	
14.	Mechanism for retrieving frozen or NSQ medical products	Communication with the department like Labour room, emergency etc<br><br>Medical products retrieval documentation	
15.	Segregation of frozen or NSQ Medical Products	Availability of Identified area for separating for frozen or NSQ Medical Products<br><br>Frozen or NSQ medical products reflected in the store/ stock register	
16.	Ensuring the right medical products for dispensing to the patient	Verification at two level-<br><br>During prescription reading<br><br>During the dispensing	
17.	Adequate auxiliary labelling of medical products before dispensing to the patient	Materials used for additional -labelling- sticker, marker etc.<br><br>Patient name Dosage, Shake well before use, For external use, etc.	
18.	Medication Counselling	Storage, dosage, frequency, timing,	

		way/method of usage/administration, route of administration, drug interaction/ compatibility, adverse drug reaction, diet and lifestyle modifications	
19.	Patient Queries	Listening calmly<br><br>Respond in language understandable to the patient<br><br>Information-Correct, Complete,<br><br>Keep it short and simple (KISS)	
20.	Mechanism to return unused, unwanted, or expired medical products to Pharmacy	Information shared during dispensing<br><br>Recording of returned medical products	
21.	Communication	A. Active listening<br>B. Verbal Communication-Clear, Precise<br>C. Non-verbal communication<br>D. Written Communication-Check documents like – Leave application; Indenting to Procure required Items etc.<br>E. Use of terms understandable to the patient<br>F. Cultural awareness and sensitivity<br>G. Responsiveness towards Patient queries including medical products	
22.	Effective interdisciplinary/intra professional Communication	Observe his/her interdisciplinary/intra professional Communication.<br><br>Whether he fulfils his role in the medical team	
23.	Official Communication	Content should be<br>• Complete,<br>• Clear<br>• Precise	

C.3. Mini Clinical Evaluation of selected Conditions (Skills)

Score Definition

A. None—No demonstrated skills at all/does not perform the task(s) completely
B. Limited Demonstrated very limited strengths/skills in this area
C. Some—Demonstrated some ability/skills in this area.
D. Strong—Demonstrated strong skills/strength in this area.
E. Excellent—Demonstrated excellent skills/strength in this area.
F. Not applicable
G. Don't know- Not even heard about that skill
H. Skill limitation is clearly related to resource limitations

Domain Code	S.N.	Evaluation Points	Means of Verification	Response (Score)	Remarks
1	1.	Inventory Management Technique (VED)	Verification of medicines given<br><br>Grouping them into three categories<br><br>Action taken		
	2.	Appropriately Organizing the medical products according to the storage need	Matching with the product and recommended storage condition.		
	3.	Medical Products Identification based on epidemiological need of Catchment Population	Identification of the disease<br><br>Understanding the Standard Treatment Guideline/ workflows for the identified disease.		
	4.	Forecasting the requirements of Medical Products-Based on the past consumption	Past Consumption Data taking consideration of stock out period<br><br>Calculation for the requirement of medical Products taking account of population growth or epidemiological transition for		

			two years.		
	5.	Local Purchasing Documentation	Able to fill the Standard Formats<br><br>Preservation of Purchasing documents<br><br>Any additional documentation required		
	6.	Contingency Plan for Stock out	Local Purchasing procedures in Place<br><br>Facilitate the supplies from neighboring institutions or district store.		
	7.	Verification Checklist for receiving supplies	Is the Pharmacist able to prepare a checklist for receiving the supplied identified medical products?<br><br>-Physical Check for Quantity and damage		
	8.	Status Tracking for Ordered supplies	**If the MIS is available for Supply Chain-**<br>Ability to check the delivery status (expected date)<br><br>**If the MIS is not available for supply chain**-<br>Able to communicate and ascertain the status of delivery		
	9.	Alternate Procurement Procedure	**Local Purchase**<br>Able to fill the Standard Formats		

			Preservation of Purchasing documents<br><br>Any additional documentation required<br>**Inter institutional Transfer**<br><br>Knowledge about the availability of required medical products in other institutions<br><br>Documentation for Medical Products transfer<br><br>Mechanism to transfer the medical products to your institutions		
	10.	SOP Development	-Identification of processes like, Stock Verification		
	11.	Identification of Slow-moving medical products and corrective action to be required	Verification of Stock register<br><br>Alerting the medical officer to inform about the slow-moving medical products to the higher centers.		
	12.	Preparation for attending any meeting	Understanding agenda<br><br>Based on Agenda, prepare the note for the points, needs to express during the meeting		
	13.	Risk Management Plan	In the event of Fire accident, what is your SOP?		
	14.	Use of Microsoft Office Package- Word, Excel &	-Create file for Word, Excel &		

		Power point	Power point<br><br>- Prepare a document in Microsoft Word, excel and Power point		
	15.	Stock Verification	Each item of pharmacy stock register needs to be verified physically through records of procurement, dispensing refund, indent/ issue, stock transfers, & stock taken last month.<br><br>The list for stock-taking has the following particulars:<br><br>*Name of the medicine, Quantity, Batch number, Expiry date, Manufacturing date, Manufacturer Name, Quantity counted, Quantity as per records, Quantity as per store, Discrepancy (if any), Discrepancy after reconciliation, Signature of the Pharmacist;*		
3	16.	Prescription Validation	Facility Name<br><br>Medical Officer signature / initial		
	17.	Reporting system for dispensing errors and near misses	Reporting Elements<br><br>Name of the Reporter		
	18.	Monitoring Medication Adherence for Chronic diseases	Pill Counting<br><br>Medication Refill Rate		
	19.	Adverse Drug Reaction (ADR) - Events reporting	Form Availability<br><br>Fill form for Adverse drug		

			reaction events		
4	20.	Bio Medical Waste Management – SOP for Outreach Camps	**Important Components** –<br>Collection<br>Segregation<br>Transportation		
	21.	Listing of emergency medicines during disaster management	Listing of Common illness/ health conditions-Fever, Diarrhea etc.<br><br>Identification of medicines for the identified common illness/ health conditions		
	22.	First Aid	Condition-Wound management		
	23.	Cardiopulmonary Resuscitation	How to perform?<br>Steps-<br>A. Check the area is safe<br>B. Shake and shout<br>C. Call for help<br>D. Open airway by placing one hand on the forehead and the other 2 fingers under the chin<br>E. Check for breathing for 10 seconds<br>F. Clear chest & give 30 compressions with 2 hands, fingers and elbows locked, hands clasped<br>G. Close nose, open mouth, breathe into casualty's mouth, turn your head & breathe again<br>H. Repeat until help arrives		

10	24.	Carry out basic tests/ measurements	Blood Pressure<br>Hemoglobin<br>BMI Calculation<br>Pregnancy Test		
	25.	Assemble/fit, use Oxygen cylinder, along with administration of oxygen			
	26.	Nebulization			
	27.	Wound Management	Cleaning<br>Medicine Application or use<br>Bandaging		
	28.	Diagnosis- Malaria	Signs<br>Symptoms<br>Rapid Diagnostic Tests		
	29.	Diagnosis- Upper Respiratory Tract Infection	Signs<br>Symptoms		
	30.	Diagnosis- Scabies	Signs<br>Symptoms		

C.4. Simulation Exercise

Domain Code	Case	Response (Satisfactory/ Unsatisfactory)	Remarks
1	**Timely Corrective measures**<br>Assume that tomorrow you come to Pharmacy and engaged in your dispensing of medical products. At 11 :00 AM, you received the demand of Tetanus Toxoid from Medical Officer In charge for treating accident case. For which, you opened the refrigerator and found that refrigerator is out of order. In this condition, what would be your		

	immediate plan of corrective action?		
	**Reordering Time**<br><br>Suppose the monthly consumption of Gentamycin Injection is 50 vials. During summer season, you get to know that the possible serious bacterial infection in young infants has been rose more than three times. Considering this episode of sudden rise in cases, what are the parameters you will consider before making indenting to meet the demand of extra Gentamycin vials?		Delivery Time and Quantity
	**Suggest Medical officer to alter medicine**<br><br>Assume that tomorrow you encounter stock out of Amoxycillin in your facility. In this case, which alternative medicine, you will prefer to suggest your medical officer?		Azithromycin
	**Possible Drug-Drug/Drug-Food Interaction:**<br><br>Iron and Calcium-<br><br>Assume tomorrow afternoon, a pregnant woman, comes to you with a prescription for Iron Folic Acid Tablet and Calcium Tablet. In the prescription, it mentions that both the tablets to be taken on daily basis. In this case, that is the possibility that the patient takes the medicine together. So, to avoid this situation of drug-drug interaction, what do you suggest?<br><br>Iron and Food-<br><br>Suppose that a woman comes to you with a complaint that she is feeling nauseating after taking Iron tablet. On enquiry, you find that the woman is taking Iron tablet in empty stomach. So, for this condition, what do you advise to the woman?		The time interval for taking these medicines should be at least 2 Hours. Through this interval, we may reduce the possible Drug-Drug interaction.<br><br>Taking Iron tablet after food will reduce iron absorption but it will promote adherence.

Bilingual Competency Assessment Tool

Competency Assessment Tool- Pharmacists
ଦକ୍ଷତା ମୂଲ୍ୟାୟନ ପ୍ରଶ୍ନାବଳି -ଫାର୍ମାସିଷ୍ଟ

A. General Information:
A. ସାଧାରଣ ସୂଚନା:

A1. Date & Time:<br>A1. ତାରିଖ ଏବଂ ସମୟ:	A4. Designation:<br>A4. ପଦବୀ:
A2. Age:<br>A2. ବୟସ:	A5. Health Facility:<br>A5. ସ୍ୱାସ୍ଥ୍ୟ ସେବାସ୍ଥଳ:
A3. Gender:<br>A3. ଲିଙ୍ଗ:	A6. District:<br>A6. ଜିଲ୍ଲା:

A7. Education & In-Service Training:<br>A7. ଶିକ୍ଷା ଏବଂ ସେବାକାଳୀନ ପ୍ରଶିକ୍ଷଣ:		Pharmacy Education<br>ଫାର୍ମାସି ଶିକ୍ଷା	In-Service Trainings<br>ସେବାକାଳୀନ ପ୍ରଶିକ୍ଷଣ	Others Education<br>ଅନ୍ୟାନ୍ୟ ଶିକ୍ଷା
	Name of Institution/ Agencies<br>ଅନୁଷ୍ଠାନ/ଏଜେନ୍ସିର ନାମ			
	Year of Completion<br>ସମ୍ପୂର୍ଣ୍ଣ ହେବାର ବର୍ଷ			

A8. Postings details<br>A8. କାର୍ଯ୍ୟସ୍ଥଳର ବିବରଣୀ	Place of Posting<br>କାର୍ଯ୍ୟସ୍ଥଳର ଅବସ୍ଥାନ	District<br>ଜିଲ୍ଲା	Period (From-to)<br>ଅବଧି (ଠାରୁ-ପର୍ଯ୍ୟନ୍ତ)	Regular/ Contractual<br>ନିୟମିତ/ଠିକା	Reason(s) for transfer<br>ବଦଳି ହେବାର କାରଣ(ଗୁଡ଼ିକ)
1					
2					
3					
4					
5					

B. **General Questions (Self Appraisal):**

B. ସାଧାରଣ ପ୍ରଶ୍ନାବଳି (ନିଜେ ମୂଲ୍ୟାୟନ କରିବେ):

B1. Do you know how many drugs are provisioned (stored and supplied) for your facility?<br><br>B1. ଆପଣ ଜାଣନ୍ତି କି ଆପଣଙ୍କ ଡାକ୍ତରଖାନା ପାଇଁ କେତେ ପ୍ରକାରର ଔଷଧର ଯୋଗାଇ ଦିଆଯାଇଥାଏ (ଯାହାକି ଗଚ୍ଛିତ ରହିଛି ଏବଂ ଯୋଗାଇ ଦିଆଯାଏ)?
B2. In your sense, out of provisioned drugs (stored and supplied) for your facility, currently, roughly, how many drugs are available?<br>B2. ଆଚ୍ଛା ଆପଣ ଅନୁମାନ କରି କହିଲେ ଆପଣଙ୍କ ଡାକ୍ତରଖାନାରେ ଯେତିକି ଔଷଧ ଯୋଗାଇ ଦେବାର ବ୍ୟବସ୍ଥା ରହିଛି (ଆପଣଙ୍କ ଷ୍ଟୋରରେ ରହିଥାଏ) ତାହା ମଧ୍ୟରୁ କେତେ ପ୍ରକାର ଏବେ ଆପଣଙ୍କ ପାଖରେ ରହିଛି?
B.3. What are the other daily activities, apart from Pharmacy or drug related?<br><br>B.3. ଫାର୍ମାସି ବା ଔଷଧ ସମ୍ପର୍କିତ କାମ ସହିତ ଆପଣ ପ୍ରତିଦିନ ଆଉ କି କି କାମ କରିଥାନ୍ତି?
B4. In your opinion, how competent are you in performing your day-to-day activities?<br><br>B4. ଆପଣଙ୍କ ମତରେ ନିତିଦିନିଆ କାମରେ ଆପଣ କେତେ ଦକ୍ଷ?
B5. On a scale of 1-5, how would you rate your overall knowledge & skills to perform your duty?<br><br>(1-very little, 2- less, 3- adequate, 4- more than needed, 5-exceptional)<br>B5. 1ରୁ 5 ପର୍ଯ୍ୟନ୍ତର ସ୍କେଲରେ, ଆପଣଙ୍କର କାମ ବିଷୟରେ ଆପଣଙ୍କର ମୋଟାମୋଟି ଜ୍ଞାନ ଓ ଦକ୍ଷତାକୁ ଆପଣ କେତେ ନମ୍ବର ଦେବେ?<br>(1- ଖୁବ୍ କମ୍, 2- କମ୍, 3- ଯଥେଷ୍ଟ, 4 - ଆବଶ୍ୟକଠାରୁ ଅଧିକ, 5 - ଅସାଧାରଣ)
B6. What skills do you have that are most useful in carrying out the assigned duties efficiently and effectively?<br><br>B6. ଆପଣଙ୍କର କେଉଁ ଦକ୍ଷତା ଯୋଗୁଁ ଆପଣଙ୍କୁ ଦିଆଯାଇଥିବା କାମକୁ ଆପଣ ଠିକ୍ ଭାବରେ ଯଥା ସମୟରେ କରିପାରନ୍ତି?

B7. Do you feel you have additional skills to perform tasks other than your assigned duties?<br><br>B7. ଆପଣଙ୍କୁ ଦିଆଯାଉଥିବା କାମ ସହିତ ଆପଣ ଆଉ କିଛି କାମ କରିବା ପାଇଁ ଦକ୍ଷ ବୋଲି ଭାବୁଛନ୍ତି କି?
B8. What have all pieces of in-service training been given to you to make yourself competent to carry out the assigned duties?<br><br>B8. ଆପଣଙ୍କୁ ଦିଆଯାଉଥିବା କାମକୁ ଠିକ୍ ଭାବରେ କରିବା ପାଇଁ ଆପଣଙ୍କୁ କାମ ବିଷୟରେ ସମୟ ସମୟରେ କିଛି ତାଲିମ୍ ଦିଆଯାଉଛି କି?
B9. Is there any mechanism to provide training/ orientation before introducing new process (like digital platforms)?<br><br>B9. କୌଣସି ନୂଆ ଢଙ୍ଗରେ କାମ କରିବା ଆରମ୍ଭ କରିବା ପୂର୍ବରୁ ଏହା ସହିତ ପରିଚିତ ହେବା ପାଇଁ/ ଆଗୁଆ ତାଲିମ୍ ଦେବାର କୌଣସି ବ୍ୟବସ୍ଥା ରହିଛି କି (ଯେପରିକି ଡିଜିଟାଲ୍ ପ୍ଲାଟଫର୍ମଗୁଡ଼ିକ)?
B10. In your opinion, what are additional pieces of training required for you to carry out your duties efficiently and effectively?<br><br>B10.ଆଚ୍ଛା କହିଲେ ଆପଣ କାମକୁ ଆହୁରି ଭଲ ଭାବରେ ତଥା ସହଜରେ କରିବା ପାଇଁ ଅଧିକ ତାଲିମ୍ ଦରକାର ବୋଲି ଭାବୁଛନ୍ତି କି?
B11. Do you feel that you are performing well or at least at par with your expectations in this job?<br>If yes/no, then why do you feel so?<br><br>B11. ଆପଣ ଭାବୁଛନ୍ତି କି ଆପଣଙ୍କ କାମ ଠିକ ଭାବରେ କରୁଛନ୍ତି ବା ଅତିକମ୍‌ରେ ଆପଣଙ୍କଠାରୁ ଯାହା ଆଶା କରାଯାଉଛି ସେତିକି ତ ଆପଣ କରିପାରୁଛନ୍ତି?<br>ଯଦି ହଁ/ନା, ତେବେ ଆପଣ କାହିଁକି ଏପରି ଭାବୁଛନ୍ତି?
B12. How would you describe your working conditions here at this facility?<br><br>B12. ଏହି ଡାକ୍ତରଖାନାରେ ଆପଣଙ୍କ କାର୍ଯ୍ୟ ପରିବେଶକୁ ଆପଣ କିପରି ବର୍ଣ୍ଣନା କରିବେ?
B13. What working conditions favours /distracts, you from carrying out your duties?<br><br>B13. ଆପଣଙ୍କ ଡାକ୍ତରଖାନାରେ କେଉଁ କାର୍ଯ୍ୟସ୍ଥିତି ଯୋଗୁଁ ଆପଣଙ୍କୁ କାମ କରିବାକୁ ଭଲ ଲାଗେ ବା କେଉଁ କାର୍ଯ୍ୟସ୍ଥିତି ଯୋଗୁଁ ଆପଣଙ୍କ କାମରେ ମନ ଲାଗେ ନାହିଁ?
B 14. What is the supply chain mechanism followed to ensure the availability of adequate drugs and consumables, in your facility?<br><br>B 14. ଆପଣଙ୍କ ଡାକ୍ତରଖାନାରେ ଯଥେଷ୍ଟ ପରିମାଣର ଔଷଧ ଏବଂ ବ୍ୟବହାର ହେଉଥିବା ଜିନିଷ ମହଜୁଦ ରହିବା ସୁନିଶ୍ଚିତ କରିବା ପାଇଁ କେଉଁ ଯୋଗାଣ ଶୃଙ୍ଖଳ ବ୍ୟବସ୍ଥା ଅନୁସରଣ କରାଯାଉଛି?

Competency Assessment Section

ଦକ୍ଷତା ମୂଲ୍ୟାୟନ ବିଭାଗ

C. Pharmacists' Domains[6]
C. ଫାର୍ମାସିଷ୍ଟମାନଙ୍କର କ୍ଷେତ୍ର

Domains	Codes
Pharmacy/ Store	1
Assistance to Medical Officer in Preparation and Implementation of different Projects and Programmes	2
Dispensing Medical Products	3
Outreach to the community	4
Ensure compliance to regulations on drugs	5
Professional Practice	6
Ethical practice	7
Communication Skill	8
Workplace Management	9
Emergency Role /Clinical Role in absence of Medical Officer and Staff Nurse	10
Continuing Professional Development	11

ଡୋମେନ ବା କ୍ଷେତ୍ର	କୋଡ୍ ଗୁଡ଼ିକ
ଫାର୍ମାସି/ଷ୍ଟୋର	1
ବିଭିନ୍ନ ପ୍ରକଳ୍ପ ଏବଂ କାର୍ଯ୍ୟକ୍ରମ ପ୍ରସ୍ତୁତି ଓ କାର୍ଯ୍ୟକାରୀ କରିବାରେ ମେଡିକାଲ୍ ଅଫିସରଙ୍କର ସହାୟତା କରିବା	2
ଔଷଧପତ୍ର ବିତରଣ କରିବା	3
ଗୋଷ୍ଠୀ ନିକଟରେ ପହଞ୍ଚିବା	4
ଔଷଧ ସମ୍ପର୍କିତ ନୀତିନିୟମ ଅନୁପାଳନ ସୁନିଶ୍ଚିତ କରିବା	5
ବୃତ୍ତିଗତ ବିଧି ବ୍ୟବହାର	6
ନୈତିକତାପୂର୍ଣ୍ଣ ବିଧି ବ୍ୟବହାର	7
ଯୋଗାଯୋଗ ଦକ୍ଷତା	8
କାର୍ଯ୍ୟସ୍ଥଳ ପରିଚାଳନା	9
ମେଡିକାଲ୍ ଅଫିସର ବା ଡାକ୍ତର ଏବଂ ଷ୍ଟାଫନର୍ସଙ୍କ ଅନୁପସ୍ଥିତିରେ ଜରୁରୀକାଳୀନ ଭୂମିକା/ନୈଦାନିକ ଭୂମିକା	10
ବୃତ୍ତିଗତ ବିକାଶ ଜାରି ରଖିବା	11

This tool is meant to assess the competencies of Pharmacists in terms of **Knowledge**[7], **Skills**[8] and **Attitudes**[9] through **Questionnaire, Direct Observation of Procedural skills**, **Mini Clinical Evaluation (Selected Conditions)** and **Simulation Exercise**[10].

ଏହି ସାଧନ ପ୍ରଶ୍ନାବଳି, ପଦ୍ଧତି କରିବା ସମୟରେ ଦକ୍ଷତାର ପ୍ରତ୍ୟକ୍ଷ ଅନୁଧ୍ୟାନ, ସଂକ୍ଷିପ୍ତ ନୈଦାନିକ ମୂଲ୍ୟାୟନ **(ବଛାଯାଇଥିବା ଅବସ୍ଥାଗୁଡ଼ିକ)** ଏବଂ **ସିମୁଲେସନ୍ ଏକ୍ସରସାଇଜ୍** ମାଧ୍ୟମରେ **ଫାର୍ମାସିଷ୍ଟମାନଙ୍କର ଜ୍ଞାନ**[11], **ଦକ୍ଷତା**[12] ଏବଂ **ମନୋଭାବ**[13] **ଦୃଷ୍ଟିରୁ ସାମର୍ଥ୍ୟ ମୂଲ୍ୟାୟନ କରିବା ପାଇଁ ଉଦ୍ଦିଷ୍ଟ**[14]

C.1. Questionnaire (Knowledge and Attitude)

ପ୍ରଶ୍ନାବଳି (ଜ୍ଞାନ ଏବଂ ମନୋଭାବ)

Domains Code<br>ଡୋମେନ ବା କ୍ଷେତ୍ର କୋଡ୍	S. N.<br>କ୍ର.ସଂ.	Points<br>ପଏଣ୍ଟଗୁଡ଼ିକ	Knowledge/ Attitude/ Skill<br>ଜ୍ଞାନ/ମନୋଭାବ/ ଦକ୍ଷତା	Response (Yes/No)<br>ଜବାବ (ହଁ/ନା)	Remarks<br>ଟିପ୍ପଣୀ
1		Do you know about the availability of any guidelines (State/ National) that defines the size of Pharmacy for Primary health Centre?<br>ଆପଣ ପ୍ରାଥମିକ ସ୍ୱାସ୍ଥ୍ୟ କେନ୍ଦ୍ର ପାଇଁ ଫାର୍ମାସିର ଆକାରକୁ ବର୍ଣ୍ଣନା କରିବା ପାଇଁ କୌଣସି ନିର୍ଦ୍ଦେଶିକା (ରାଜ୍ୟ/ଜାତୀୟ ସ୍ତରୀୟ) ରହିଥିବା ବିଷୟରେ ଜାଣନ୍ତି କି?	Knowledge<br>ଜ୍ଞାନ		
		Do you know, what should be the size/area of Pharmacy of your Primary Health Care Centre?<br>ଆପଣ ପ୍ରାଥମିକ ସ୍ୱାସ୍ଥ୍ୟ କେନ୍ଦ୍ର ପାଇଁ ଫାର୍ମାସିର ଆକାର/କ୍ଷେତ୍ରଫଳ କ'ଣ ହେବା ଉଚିତ୍ ଜାଣନ୍ତି କି?	Knowledge<br>ଜ୍ଞାନ		
		Do you know about any of Pharmacy Infrastructure assessment tool (Format) ? / Do you have prepared any tool to assess Pharmacy Instructure for your facility?<br>ଆପଣ କୌଣସି ଫାର୍ମାସି ଇନଫ୍ରାଷ୍ଟ୍ରକଚର୍ ଆସେସମେଣ୍ଟ ଟୁଲ୍ ବା ଫାର୍ମାସି ଭିତ୍ତିଭୂମି ମୂଲ୍ୟାୟନ ସାଧନ (ଫର୍ମାଟ୍) ବିଷୟରେ ଜାଣନ୍ତି କି?/ ଆପଣଙ୍କ ଡାକ୍ତରଖାନାର ଫାର୍ମାସି ବ୍ୟବସ୍ଥାର ମୂଲ୍ୟାୟନ କରିବାକୁ କୌଣସି ଉପକରଣ ପ୍ରସ୍ତୁତ କରିଛନ୍ତି କି?	Knowledge<br>ଜ୍ଞାନ		

---

1 Broadest Category of Competencies
7 Concepts and Theories
8 Use of techniques to integrate knowledge into practice
9 A person's feelings, values, and beliefs, which influence their behaviour and the performance of tasks
10 A Simulation Exercise (SimEx) is a fabricated situation, similar to happening in real life.
1. ଦକ୍ଷତାର ବ୍ୟାପକ ବର୍ଗ
2. ଧାରଣା ଏବଂ ବିଷୟଗୁଡ଼ିକ
3. କାର୍ଯ୍ୟ ବିଧିରେ ଜ୍ଞାନ ସୁସଂଯୋଜିତ କରିବା ପାଇଁ ଜ୍ଞାନକୌଶଳ ବା ଟେକନିକ୍ର ବ୍ୟବହାର
4. ଜଣେ ବ୍ୟକ୍ତିଙ୍କର ଅନୁଭବ, ମୂଲ୍ୟବୋଧ ଏବଂ ବିଶ୍ୱାସ ତାଙ୍କର ଆଚରଣ ଓ କାର୍ଯ୍ୟରେ ପ୍ରଦର୍ଶନକୁ ପ୍ରଭାବିତ କରିଥାଏ
5. ଏକ ସିମୁଲେସନ୍ ଏକ୍ସସରସାଇଜ୍ (SimEx) ହେଉଛି ଏକ କଳ୍ପିତ ପରିସ୍ଥିତି ଯାହାକି ବାସ୍ତବ ଜୀବନ ସଦୃଶ ହୋଇଥାଏ।

	0.	Do you think, Infrastructure assessment helps in identifying gaps in your Pharmacy?<br>ଆପଣ ଭାବୁଛନ୍ତି କି, ଏହି ଫାର୍ମାସି ବ୍ୟବସ୍ଥାର ମୂଲ୍ୟାୟନ କରିବା ଦ୍ୱାରା ଆପଣଙ୍କ ଫାର୍ମାସିରେ ରହିଥିବା ଅସୁବିଧା ଚିହ୍ନଟ କରାଯାଇପାରିବ?	Attitude<br>ମନୋଭାବ		
	1.	In your sense, if the identified gaps get fulfilled in your Pharmacy, can lead to better performance of your Pharmacy?<br>ଆପଣ ଭାବୁଛନ୍ତି କି ଯଦି ଚିହ୍ନଟ କରାଯାଇଥିବା ଅସୁବିଧାଗୁଡ଼ିକ ପୂରଣ କରି ଦିଆଯାଏ, ଆପଣଙ୍କ ଫାର୍ମାସି ଅଧିକ ଦକ୍ଷତାର ସହ କାର୍ଯ୍ୟ କରିପାରିବ?	Attitude<br>ମନୋଭାବ		
	2.	Do you think, the inventory management techniques could support the pharmacist in managing the medical products better?<br>ଆପଣ ଭାବୁଛନ୍ତି କି, ଇନଭେଣ୍ଟୋରୀ ପରିଚାଳନା ଜ୍ଞାନକୌଶଳ ଫାର୍ମାସିଷ୍ଟମାନଙ୍କୁ ଚିକିତ୍ସାରେ ବ୍ୟବହୃତ ସାମଗ୍ରୀ ଠିକ୍ ଭାବରେ ପରିଚାଳନା କରିବାରେ ସାହାଯ୍ୟ କରିବ?	Attitude<br>ମନୋଭାବ		
	3.	Do you know about the parameters for segregation of various products in the pharmacy and pharmacy store including the quarantine place?<br>ଆପଣ ଫାର୍ମାସିରେ ଏବଂ ଫାର୍ମାସି ଷ୍ଟୋରରେ {କ୍ୱାରେଣ୍ଟାଇନ୍ (Quarentine) ସ୍ଥାନକୁ ଅନ୍ତର୍ଭୁକ୍ତ କରି} ବିଭିନ୍ନ ଜିନିଷ ଅଲଗା ଅଲଗା କରି ରଖିବା ପାଇଁ ମାନଦଣ୍ଡଗୁଡ଼ିକ ବିଷୟରେ ଜାଣନ୍ତି କି?	Knowledge<br>ଜ୍ଞାନ		
	4.	Do you think, the labelling of segregated places for medical products is a useful exercise?<br>ଆପଣ ଭାବୁଛନ୍ତି କି, ଚିକିତ୍ସାରେ ବ୍ୟବହୃତ ସାମଗ୍ରୀଗୁଡ଼ିକ ପାଇଁ ଧାର୍ଯ୍ୟ ହୋଇଥିବା ଅଲଗା ଅଲଗା ସ୍ଥାନଗୁଡ଼ିକରେ ଲେବଲ୍ ଲଗାଇବା ଦରକାର କି?	Attitude<br>ମନୋଭାବ		
	5.	Do you know that the pest control is also essential component of Good Storage practice in Pharmacy and pharmacy store?<br>ଫାର୍ମାସି ଏବଂ ଫାର୍ମାସି ଷ୍ଟୋରରେ ଜିନିଷ ସୁରକ୍ଷିତ ରଖିବା ପାଇଁ କୀଟପତଙ୍ଗ ନିୟନ୍ତ୍ରଣ (ପେଷ୍ଟ କଣ୍ଟ୍ରୋଲ୍) ଆବଶ୍ୟକ ହୋଇଥାଏ ବୋଲି ଆପଣ ଜାଣନ୍ତି କି?	Knowledge<br>ଜ୍ଞାନ		
	6.	Do you know that the frequent pest control is necessary for Pharmacy and pharmacy store?<br>ଫାର୍ମାସି ଏବଂ ଫାର୍ମାସି ଷ୍ଟୋରରେ ନିୟମିତ ବ୍ୟବଧାନରେ କୀଟପତଙ୍ଗ ନିୟନ୍ତ୍ରଣ (ପେଷ୍ଟ କଣ୍ଟ୍ରୋଲ୍) କରାଯିବା ଆବଶ୍ୟକ ବୋଲି ଆପଣ ଜାଣନ୍ତି କି?	Knowledge<br>ଜ୍ଞାନ		
	7.	Have you ever observed any quality issues during storage of medical products in your pharmacy and Pharmacy store during your service?<br>ଆପଣଙ୍କ ସର୍ଭିସ୍ ସମୟରେ ଆପଣଙ୍କର ଫାର୍ମାସି ଏବଂ ଫାର୍ମାସି ଷ୍ଟୋରରେ , ଚିକିତ୍ସାରେ ବ୍ୟବହୃତ ସାମଗ୍ରୀ ସଂରକ୍ଷଣ କାର୍ଯ୍ୟରେ ଗୁଣବତ୍ତାକୁ ନେଇ କୌଣସି ସମସ୍ୟା	Attitude<br>ମନୋଭାବ		

		ରହିଥିବା ଆପଣ ଜାଣନ୍ତି କି?			
	8.	Do you think that regular checking of storage conditions (Temperature) is essentially required for medical products?<br>ଚିକିତ୍ସାରେ ବ୍ୟବହୃତ ସାମଗ୍ରୀ ପାଇଁ ନିୟମିତ ଭାବରେ ସଂରକ୍ଷଣ ସ୍ଥିତି (ତାପମାତ୍ରା) ଯାଞ୍ଚ କରିବା ଆବଶ୍ୟକ ବୋଲି ଆପଣ ଭାବୁଛନ୍ତି କି?	Attitude<br>ମନୋଭାବ		
	9.	Would you like to use the medical products whose appearance has been changed during the storage?<br>ଚିକିତ୍ସାରେ ବ୍ୟବହୃତ ଯେଉଁ ସାମଗ୍ରୀର ରୂପ, ରଙ୍ଗ ବା ଗଠନ ସଂରକ୍ଷଣ ସମୟରେ ବଦଳିଯାଇଥାଏ, ଆପଣ ତାହାକୁ ବ୍ୟବହାର କରିବେ କି?	Attitude<br>ମନୋଭାବ		
	0.	Do you think, the storage of medical products in safe and secure manner could help the pharmacist in reducing pilferages?<br><br>ଚିକିତ୍ସାରେ ବ୍ୟବହୃତ ସାମଗ୍ରୀକୁ ସୁରକ୍ଷିତ ଭାବରେ ଏବଂ ନିରାପଦରେ ସାଇତି ରଖିବା ଫାର୍ମାସିଷ୍ଟଙ୍କୁ ଜିନିଷ ନଷ୍ଟ ହେବା କମ୍ କରିବାରେ ସାହାଯ୍ୟ କରିପାରିବ କି?	Attitude<br>ମନୋଭାବ		
	1.	Do you know about the methodology for forecasting the requirement of medical products?<br>ଚିକିତ୍ସାରେ ବ୍ୟବହୃତ ସାମଗ୍ରୀଗୁଡ଼ିକର ଆବଶ୍ୟକତା ବିଷୟରେ କିପରି ଆଗୁଆ ଅନୁମାନ କରିବେ ଜାଣନ୍ତି କି?	Knowledge<br>ଜ୍ଞାନ		
	2.	Do you feel the need of forecasting of requirements for medical products in your facility?<br><br>ଆପଣଙ୍କ ଡାକ୍ତରଖାନାରେ ଚିକିତ୍ସାରେ ବ୍ୟବହୃତ ସାମଗ୍ରୀର ଆବଶ୍ୟକତା ବିଷୟରେ ଆଗୁଆ ଅନୁମାନ କରିବା ଦରକାର ହୋଇଥାଏ କି?	Attitude<br>ମନୋଭାବ		
	3.	Do you know about the local purchasing rules for medical products?<br>ଆପଣ ଚିକିତ୍ସାରେ ବ୍ୟବହୃତ ସାମଗ୍ରୀଗୁଡ଼ିକ ସ୍ଥାନୀୟ ଭାବରେ କିଣିବା ପାଇଁ ନିୟମାବଳି କ'ଣ ରହିଛି ଜାଣନ୍ତି କି?	Knowledge<br>ଜ୍ଞାନ		
	4.	Do you know how to document the local purchasing of medical products?<br>ଆପଣ ସ୍ଥାନୀୟ ଭାବରେ ଚିକିତ୍ସାରେ ବ୍ୟବହୃତ ସାମଗ୍ରୀ ଗୁଡ଼ିକର କିଣିବା ବିବରଣୀ କିପରି ଲିପିବଦ୍ଧ କରିବେ ଜାଣନ୍ତି କି?	Knowledge<br>ଜ୍ଞାନ		
	5.	Do you think the documentation is important in the case of local purchasing of medical products?<br><br>ଚିକିତ୍ସାରେ ବ୍ୟବହୃତ ସାମଗ୍ରୀ ସ୍ଥାନୀୟ ଭାବରେ କିଣାକିଣି କରିବା ବିବରଣୀ ଲିପିବଦ୍ଧ କରିବା ଆବଶ୍ୟକ ହୋଇଥାଏ	Attitude<br>ମନୋଭାବ		

		କି?			
	6.	Do you prefer to engage healthcare providing team members of your facility before preparing the indent?<br><br>ଆପଣଙ୍କ ଡାକ୍ତରଖାନାରେ ଇଣ୍ଡେଣ୍ଟ (Indent) ପାଇଁ ପ୍ରସ୍ତୁତ ହେବା ପୂର୍ବରୁ ସ୍ୱାସ୍ଥ୍ୟ ଯତ୍ନ ପ୍ରଦାନକାରୀ ଦଳର ସଦସ୍ୟମାନଙ୍କୁ ଯେପରି କି ନର୍ସମାନଙ୍କୁ ସାମିଲ୍ କରିବାକୁ ଚାହିଁବେ କି?	Attitude<br>ମନୋଭାବ		
	7.	Do you feel that the contingency plan can help the pharmacist in reducing stock out of medical products?<br><br>କଣ୍ଟିଜେନ୍ସି ପ୍ଲାନ୍ ବା ଆକସ୍ମିକ ପରିସ୍ଥିତି ପାଇଁ ଯୋଜନା ଫାର୍ମାସିଷ୍ଟମାନଙ୍କୁ ଚିକିତ୍ସାରେ ବ୍ୟବହୃତ ସାମଗ୍ରୀ ର ଷ୍ଟକ୍ ସରିଯିବା ପରିସ୍ଥିତି ଏଡ଼ାଇବାରେ ସାହାଯ୍ୟ କରିପାରିବ?	Attitude<br>ମନୋଭାବ		
	8.	Do you know the concept of conflict of Interest?<br>ଆପଣ ବ୍ୟକ୍ତିଗତ ସ୍ୱାର୍ଥ ସାଧନକୁ ଦୃଷ୍ଟିରେ ରଖି (conflict of Interest) କାମ କରିବା ବିଷୟରେ ଜାଣନ୍ତି କି?	Knowledge<br>ଜ୍ଞାନ		
	9.	Do you prefer to give declaration of the non-conflict of interest for local purchasing of medical products?<br><br>ଆପଣ ଚିକିତ୍ସାରେ ବ୍ୟବହୃତ ସାମଗ୍ରୀଗୁଡ଼ିକ ସ୍ଥାନୀୟ ଭାବରେ କିଣାଯିବା କାହା ସ୍ୱାର୍ଥ ପାଇଁ କରାଯାଇ ନାହିଁ ଏହା ଘୋଷଣା କରିବାକୁ ଚାହିଁବେ କି?	Attitude<br>ମନୋଭାବ		
	0.	Do you know what the right time would be to make indent (Reorder)?<br>ଆପଣ ଇଣ୍ଡେଣ୍ଟ (ପୁନଃ ଅର୍ଡର) ପାଇଁ ସଠିକ ସମୟ କ'ଣ ଜାଣନ୍ତି କି?	Knowledge<br>ଜ୍ଞାନ		
	1.	Do you know how much time usually it requires after raising indent to get medical products delivered to your facility?<br>ଆପଣଙ୍କ ଡାକ୍ତରଖାନାରେ ଇଣ୍ଡେଣ୍ଟ ଉପସ୍ଥାପନ କରିବା ପରେ ଚିକିତ୍ସାରେ ବ୍ୟବହୃତ ସାମଗ୍ରୀ ପହଞ୍ଚିବା ପାଇଁ ସାଧାରଣତଃ କେତେ ସମୟ ଲାଗିଥାଏ?	Knowledge<br>ଜ୍ଞାନ		
	2.	Do you prefer to consider the usual delivery time (Ordering to receiving) before making an indent for medical products?<br>ଚିକିତ୍ସାରେ ବ୍ୟବହୃତ ସାମଗ୍ରୀ ପାଇଁ ଏକ ଇଣ୍ଡେଣ୍ଟ (Indent) କରିବା ପୂର୍ବରୁ ସାଧାରଣତଃ ଡେଲିଭରି ସମୟ (ଅର୍ଡର ଦେବାଠାରୁ ଯୋଗାଣ ପର୍ଯ୍ୟନ୍ତ) ବିଷୟରେ ଆପଣ ଚିନ୍ତା କରିଥାନ୍ତି କି?	Attitude<br>ମନୋଭାବ		
	3.	Do you know the alternative medicines in case of stock out?<br><br>ଷ୍ଟକ୍ ସରିଯିବା ପରିସ୍ଥିତିରେ ଏହା ପରିବର୍ତ୍ତେ ବ୍ୟବହାର	Knowledge<br>ଜ୍ଞାନ		

		ହେଉଥିବା ଔଷଧଗୁଡ଼ିକ ବିଷୟରେ ଆପଣ ଜାଣନ୍ତି କି?				
	4.	Do you prefer to suggest medical officers to alter the prescription in case of stock out? ଷ୍ଟକ୍ ସରିଯିବା ପରିସ୍ଥିତିରେ ଡାକ୍ତରଙ୍କୁ ଡାକ୍ତରୀ ଚିଠା ବଦଳାଇବା ପାଇଁ ଅନୁରୋଧ କରିବାକୁ ଆପଣ ଚାହିଁବେ କି?	Attitude ମନୋଭାବ			
	5.	Do you know which habit-forming drugs are available in your facility?<br><br>ଆପଣଙ୍କ ଡାକ୍ତରଖାନାରେ ଅଭ୍ୟାସ ସୃଷ୍ଟି କରୁଥିବା କି କି ଔଷଧ ରହିଛି ଜାଣନ୍ତି କି?	Knowledge ଜ୍ଞାନ			
	6.	Do you know how to track the use of habit-forming medical products?<br><br>ଅଭ୍ୟାସ ସୃଷ୍ଟି କରୁଥିବା ଔଷଧଗୁଡ଼ିକର ବ୍ୟବହାରର ହିସାବ କିପରି ରଖିବେ (ଟ୍ରାକ କରିବେ) ଆପଣ ଜାଣନ୍ତି କି?	Knowledge ଜ୍ଞାନ			
	7.	Are you willing to take preventive measures for safeguarding habit-forming medical products? ଅଭ୍ୟାସ - ସୃଷ୍ଟି କରୁଥିବା ଔଷଧଗୁଡ଼ିକ ପାଇଁ ବିଶେଷ ସାବଧାନତା ଗ୍ରହଣ କରିବାକୁ ଆପଣ ଚାହାଁନ୍ତି କି?	Attitude ମନୋଭାବ			
	8.	Do you know which are slow moving medical products in your facility? ଆପଣଙ୍କ ଡାକ୍ତରଖାନାରେ ଅଳ୍ପ ବ୍ୟବହାର ହେଉଥିବା ଚିକିତ୍ସାରେ ବ୍ୟବହୃତ ସାମଗ୍ରୀଗୁଡ଼ିକ ବିଷୟରେ ଜାଣନ୍ତି କି?	Knowledge ଜ୍ଞାନ			
	9.	Do you prefer to take efforts to prevent overstocking of slow-moving medical products? ଆପଣ ଅଳ୍ପ ବ୍ୟବହାର ହେଉଥିବା ଚିକିତ୍ସାରେ ବ୍ୟବହୃତ ସାମଗ୍ରୀଗୁଡ଼ିକ ଅଧିକ ପରିମାଣରେ ଷ୍ଟକ୍‌ରେ ନ ରଖିବାକୁ ଚେଷ୍ଟା କରନ୍ତି କି?	Attitude ମନୋଭାବ			
	0.	Do you prefer to attend the meeting of various committee of which Pharmacist is a member like annual indent committee, purchase committee, Condemnation committee and Prescription audit committee, RKS, Purchase committee, Infection control committee, quality assurance committee, IDSP meetings?<br><br>ଆପଣ ବିଭିନ୍ନ କମିଟିର ବୈଠକଗୁଡ଼ିକରେ ଯୋଗଦେବାକୁ ଚାହାନ୍ତି କି ଯେଉଁଠାରେ ଫାର୍ମାସିଷ୍ଟ ଜଣେ ସଦସ୍ୟ ହୋଇଥାନ୍ତି ଯେପରିକି ବାର୍ଷିକ ଇଣ୍ଡେଣ୍ଟ କମିଟି, ପର୍ଚେଜ୍ ବା କ୍ରୟ କମିଟି, କଣ୍ଡେମନେସନ୍ କମିଟି ଏବଂ ପ୍ରେସକ୍ରିପସନ୍ ଅଡିଟ୍ କମିଟି, ଆରକେଏସ୍, କ୍ରୟ କମିଟି, ସଂକ୍ରମଣ ନିୟନ୍ତ୍ରଣ କମିଟି, ଗୁଣବତ୍ତା ଆଶ୍ୱାସନା କମିଟି, ଆଇଡିଏସପି ବୈଠକଗୁଡ଼ିକ?	Attitude ମନୋଭାବ			
	1.	Do you prefer to disseminate the minutes of the meeting with the concerned persons, if required?	Attitude ମନୋଭାବ			

		ଯଦି ଆବଶ୍ୟକ ହୁଏ ଆପଣ ଏହି କାର୍ଯ୍ୟରେ ଜଡିତ ଥିବା ବ୍ୟକ୍ତିମାନଙ୍କୁ ବୈଠକର ସାରାଂଶ ଜଣାଇବାକୁ ଚାହିଁବେ କି?			
	2.	Do you know about the circulars/government orders for I.T. based Drugs and vaccines management system?<br>ଆଇଟିଭିତ୍ତିକ ଔଷଧ ଏବଂ ଟିକା ପରିଚାଳନା ବ୍ୟବସ୍ଥା ବିଷୟରେ ବିଜ୍ଞପ୍ତି/ସରକାରୀ ନିର୍ଦ୍ଦେଶଗୁଡ଼ିକ ବିଷୟରେ ଆପଣ ଜାଣନ୍ତି କି?	Knowledge<br>ଜ୍ଞାନ		
	3.	Are you interested in attending training on Drugs and vaccines management system?<br>ଔଷଧ ଏବଂ ଟିକା ପରିଚାଳନା ବ୍ୟବସ୍ଥା ଉପରେ ପ୍ରଶିକ୍ଷଣରେ ଯୋଗ ଦେବାକୁ ଆପଣ ଆଗ୍ରହୀ ହେବେ କି?	Attitude<br>ମନୋଭାବ		
	4.	In your sense, do you believe that Drugs and vaccines management system can improve pharmacist's performance?<br>ଔଷଧ ଏବଂ ଟିକା ପରିଚାଳନା ବ୍ୟବସ୍ଥା ବା ମ୍ୟାନେଜମେଣ୍ଟ ସିଷ୍ଟମ ଫାର୍ମାସିଷ୍ଟଙ୍କର କାର୍ଯ୍ୟଦକ୍ଷତାରେ ଉନ୍ନତି ଆଣିପାରିବ ବୋଲି ଆପଣ ଭାବୁଛନ୍ତି କି?	Attitude<br>ମନୋଭାବ		
	5.	Do you know how to resolve the issues/ doubts encountered by other users of E-NIRAMAYA?<br>ଅନ୍ୟ ଔଷଧ ଏବଂ ଟିକା ପରିଚାଳନା ବ୍ୟବସ୍ଥା (ଇ ନିରାମୟ) ବ୍ୟବହାର କରୁଥିବା ବ୍ୟକ୍ତି ସମ୍ମୁଖୀନ ହେଉଥିବା ଅସୁବିଧାର କିପରି ସମାଧାନ କରାଯିବ ଆପଣ ଜାଣନ୍ତି କି?	Knowledge<br>ଜ୍ଞାନ		
	6.	Are you willing to make SOP (If not available) for internal stock verification for your pharmacy/ store?<br>ଆପଣଙ୍କ ଫାର୍ମାସି /ଷ୍ଟୋର ପାଇଁ ଆଭ୍ୟନ୍ତରୀଣ ଷ୍ଟକ୍ ଯାଞ୍ଚ ନିମନ୍ତେ କାର୍ଯ୍ୟ ଶୈଳୀ (SOP) (ଯଦି ନଥାଏ) ତିଆରି କରିବାକୁ ଇଚ୍ଛୁକ ଅଟନ୍ତି କି?	Attitude<br>ମନୋଭାବ		
	7.	Do you know the term Not of Standard Quality (NSQ) Medical Products?<br>ଆପଣ ଚିକିତ୍ସାରେ ବ୍ୟବହୃତ ନଟ୍ ଅଫ୍ ଷ୍ଟାଣ୍ଡାର୍ଡ କ୍ୱାଲିଟି (ଏନଏସକ୍ୟୁ) ବା ମାନକ ଗୁଣବତ୍ତା ସମ୍ପନ୍ନ ନୁହେଁ ସାମଗ୍ରୀଗୁଡ଼ିକ ବିଷୟରେ ଜାଣନ୍ତି କି?	Knowledge<br>ଜ୍ଞାନ		
	8.	In the case of NSQ drugs availability in your Pharmacy or store, do you prefer to segregate it from the main stock?<br>ଆପଣଙ୍କ ଫାର୍ମାସି କିମ୍ବା ଷ୍ଟୋରରେ, ଏନଏସକ୍ୟୁ ଔଷଧଗୁଡ଼ିକ ରହିଥିବା ଘଟଣାରେ, ଆପଣ ଏହାକୁ ମୁଖ୍ୟ ଷ୍ଟକରୁ ଅଲଗା କରିବାକୁ ଚାହିଁବେ କି?	Attitude<br>ମନୋଭାବ		
	9.	Do you know how to record the condemnation and disposal processes to be followed for NSQ medical products?<br>ଚିକିତ୍ସାରେ ବ୍ୟବହୃତ ଏନଏସକ୍ୟୁ ସାମଗ୍ରୀଗୁଡ଼ିକ ପାଇଁ ଅନୁସରଣ କରାଯିବାକୁ ଥିବା କଣ୍ଡେମନେସନ୍ ଏବଂ ଡିସପୋଜାଲ୍ (condemnation and disposal	Knowledge<br>ଜ୍ଞାନ		

		processes) ପ୍ରକ୍ରିୟା କିପରି ରେକର୍ଡ କରାଯାଏ ଆପଣ ଜାଣନ୍ତି କି?			
	0.	In your sense, is it worth to record the condemnation and disposal processe s followed for NSQ medical products?<br>ଚିକିତ୍ସାରେ ବ୍ୟବହୃତ ଏନଏସକ୍ୟୁ ସାମଗ୍ରୀଗୁଡ଼ିକ ପାଇଁ ଅନୁସରଣ କରାଯିବାକୁ ଥିବା କଣ୍ଡେମନେସନ୍ ଏବଂ ଡିସପୋଜାଲ୍ (condemnation and disposal processes) ପ୍ରକ୍ରିୟା ରେକର୍ଡ କରିବା ଗୁରୁତ୍ୱପୂର୍ଣ୍ଣ ହୋଇଥାଏ କି?	Attitude<br>ମନୋଭାବ		
	1.	Do you know the mechanism to dispose the Not of Standard Quality (NSQ) medical products?<br>ଚିକିତ୍ସାରେ ବ୍ୟବହୃତ ନଟ୍ ଅଫ୍ ଷ୍ଟାଣ୍ଡାର୍ଡ କ୍ୱାଲିଟି (ଏନଏସକ୍ୟୁ) ବା ମାନକ ଗୁଣବତ୍ତା ସମ୍ପନ୍ନ ନୁହେଁ ସାମଗ୍ରୀଗୁଡ଼ିକ ନଷ୍ଟ କରିବା ବ୍ୟବସ୍ଥା ବିଷୟରେ ଆପଣ ଜାଣନ୍ତି କି?	Knowledge<br>ଜ୍ଞାନ		
2	2.	Do you know about the various National/ State Health Programs like Niramaya, RNTCP, VBDCP?<br>ନିରାମୟ, ଆରଏନଟିସିପି, ଭିବିଡିସିପି ପରି ବିଭିନ୍ନ ଜାତୀୟ/ରାଜ୍ୟସ୍ତରୀୟ ସ୍ୱାସ୍ଥ୍ୟ କାର୍ଯ୍ୟକ୍ରମ ବିଷୟରେ ଆପଣ ଜାଣନ୍ତି କି?	Knowledge<br>ଜ୍ଞାନ		
	3.	Do you know how to estimate the requirement of medical products for the health programs?<br>ଏକ ସ୍ୱାସ୍ଥ୍ୟ କାର୍ଯ୍ୟକ୍ରମ ପାଇଁ କେତେ ଚିକିତ୍ସାରେ ବ୍ୟବହୃତ ସାମଗ୍ରୀ ଆବଶ୍ୟକ କିପରି ହିସାବ କରିବେ ଆପଣ ଜାଣନ୍ତି କି?	Knowledge<br>ଜ୍ଞାନ		
3	4.	Do you prefer to make sure that the right medical product is on the way to dispensing to the patients?<br>ଆପଣ ରୋଗୀଙ୍କୁ ବିତରଣ କରିବା ପୂର୍ବରୁ ପରାମର୍ଶ ମୁତାବକ ସଠିକ ଚିକିତ୍ସାରେ ବ୍ୟବହୃତ ସାମଗ୍ରୀ ଦେଉଛନ୍ତି ସୁନିଶ୍ଚିତ କରିବାକୁ ଚାହିଁଥାନ୍ତି କି?	Attitude<br>ମନୋଭାବ		
	5.	Do you know what the government guidelines/ Standard Treatment Guidelines/ Standard Treatment Workflows are required to be referred to identify the issues in prescription?<br>ଆପଣ ପ୍ରେସକ୍ରିପସନ୍‌ରେ ସମସ୍ୟା ଚିହ୍ନଟ କରିବା ପାଇଁ ଦୃଷ୍ଟାନ୍ତ ଭାବରେ ରହିଥିବା ସରକାରୀ ମାର୍ଗଦର୍ଶିକା /ମାନକ ଚିକିତ୍ସା ମାର୍ଗଦର୍ଶିକା/ମାନକ ଚିକିତ୍ସା କାର୍ଯ୍ୟପ୍ରବାହ ବିଷୟରେ ଜାଣନ୍ତି କି?	Knowledge<br>ଜ୍ଞାନ		
	6.	Do you understand the term dispensing errors?<br>ଭୁଲ୍ ସାମଗ୍ରୀ ବିତରଣ କରିବା ଅର୍ଥ ଆପଣ ବୁଝନ୍ତି କି?	Knowledge<br>ଜ୍ଞାନ		
	7.	Do you know what are the factors which leads to dispensing errors?<br>କେଉଁ ବିଷୟଗୁଡ଼ିକ ଔଷଧ ପତ୍ର ବିତରଣରେ ତ୍ରୁଟିର କାରଣ ହୋଇଥାଏ?	Knowledge<br>ଜ୍ଞାନ		

	8.	Do you prefer to document the encountered errors in prescription and dispensing of medical products?<br>ଡାକ୍ତରୀ ଚିଠାରେ ତ୍ରୁଟି ଓ ଚିକିତ୍ସାରେ ବ୍ୟବହୃତ ସାମଗ୍ରୀଗୁଡ଼ିକର ବିତରଣରେ ତ୍ରୁଟି ହେଲେ ତାହାକୁ ଲିପିବଦ୍ଧ କରିବାକୁ ଆପଣ ଚାହିଁବେ କି?	Attitude<br>ମନୋଭାବ		
	9.	Do you prefer to fix auxiliary labelling the medical product before dispensing?<br>ଆପଣ ବିତରଣ କରିବା ପୂର୍ବରୁ ଚିକିତ୍ସାରେ ବ୍ୟବହୃତ ସାମଗ୍ରୀଗୁଡ଼ିକରେ ଅକଜିଲାରୀ ଲେବଲିଂ (ନାମପତ୍ର) ଲଗାଇବାକୁ ଚାହିଁଥାନ୍ତି କି?	Attitude<br>ମନୋଭାବ		
	0.	Do you prefer to report dispensing errors and near misses in your Pharmacy?<br>ଆପଣଙ୍କ ଫାର୍ମାସିରେ ବିତରଣ କରିବାରେ ତ୍ରୁଟି ଏବଂ ତ୍ରୁଟି ପାଖାପାଖି ଘଟଣାଗୁଡ଼ିକୁ ରିପୋର୍ଟ କରିବାକୁ ଚାହିଁବେ କି?	Attitude<br>ମନୋଭାବ		
	1.	In case of confusion, do you prefer to refer to the patient’s medical and medication use history, if available?<br>କୌଣସି ଘଟଣାରେ ସନ୍ଦେହ ହେଲେ, ଆପଣ ଯଦି ସୁବିଧା ଥାଏ ରୋଗୀଙ୍କର ଡାକ୍ତରୀ ସମସ୍ୟା ଏବଂ ଔଷଧ ବ୍ୟବହାର ଇତିହାସ ଦେଖିବାକୁ ଚାହିଁବେ କି?	Attitude<br>ମନୋଭାବ		
	2.	Do you think that the patient's queries on medication are worth to entertain?<br>ଆପଣ ଭାବୁଛନ୍ତି କି ରୋଗୀଙ୍କର ଔଷଧ ସେବନ ଉପରେ ବିଭିନ୍ନ ପ୍ରଶ୍ନର ଉତ୍ତର ଦେବା ଉଚିତ୍?	Attitude<br>ମନୋଭାବ		
	3.	Do you prefer to establish a system for collection and documentation of returned medical products for safe disposal?<br>ଆପଣ ବଳକା ଓ ଅବ୍ୟବହୃତ ଔଷଧପତ୍ର ସୁରକ୍ଷିତ ଭାବରେ ନଷ୍ଟ କରିବା ପାଇଁ ସେସବୁରର ସଂଗ୍ରହ ଏବଂ ତାହାକୁ ନଥିଭୁକ୍ତ କରିବା ପାଇଁ ଏକ ବ୍ୟବସ୍ଥା ରହିବା ଚାହାଁନ୍ତି କି?	Attitude<br>ମନୋଭାବ		
	4.	Do you know how to report adverse drug reaction events?<br>ଆପଣ ଔଷଧର ଅପ୍ରତ୍ୟାଶିତ ପ୍ରଭାବ (adverse drug reaction)ଘଟଣାଗୁଡ଼ିକ କିପରି ରିପୋର୍ଟ କରିବେ ଜାଣନ୍ତି କି?	Knowledge<br>ଜ୍ଞାନ		
	5.	Do you prefer to report the adverse drug reaction events?<br>ଆପଣ ଔଷଧର ଅପ୍ରତ୍ୟାଶିତ ପ୍ରଭାବ (adverse drug reaction) ଘଟଣାଗୁଡ଼ିକ ରିପୋର୍ଟ କରିବାକୁ ଚାହିଁବେ କି?	Attitude<br>ମନୋଭାବ		
	6.	Do you know the term health promotion?<br>ଆପଣ ହେଲ୍‌ଥ ପ୍ରମୋସନ୍ ବା ସ୍ୱାସ୍ଥ୍ୟରେ ଉନ୍ନତି ବିଷୟରେ ଜାଣନ୍ତି କି?	Knowledge<br>ଜ୍ଞାନ		
	7.	Do you know how to communicate the public for health promotion?<br>ଆପଣ ହେଲ୍‌ଥ ପ୍ରମୋସନ୍ ବା ସ୍ୱାସ୍ଥ୍ୟରେ ଉନ୍ନତି ପାଇଁ ସାଧାରଣ ଲୋକଙ୍କ ସହ କିପରି ଯୋଗାଯୋଗ କରିବେ	Knowledge<br>ଜ୍ଞାନ		

		ଜାଣନ୍ତି କି?			
	[illegible]8.	Do you know what IEC materials supplied by government available to inform public on disease/ illness prevention and health promotion?<br>ଆପଣ ରୋଗ/ଅସୁସ୍ଥତା ନିରାକରଣ ପାଇଁ ଏବଂ ହେଲ୍‌ଥ ପ୍ରମୋସନ୍ ବା ସ୍ୱାସ୍ଥ୍ୟରେ ଉନ୍ନତି ପାଇଁ ଜନସାଧାରଣଙ୍କୁ ଜଣାଇବା ନିମନ୍ତେ ସରକାରଙ୍କ ଦ୍ୱାରା ଯୋଗାଇ ଦିଆଯାଉଥିବା ଆଇଇସି (ଶିକ୍ଷା, ସୂଚନା, ପରାମର୍ଶ) ସାମଗ୍ରୀଗୁଡ଼ିକ ବିଷୟରେ ଜାଣନ୍ତି କି?	Knowledge<br>ଜ୍ଞାନ		
	[illegible]9.	Do you prefer to participate, as a trainer, in training community staff and leaders on disease/ illness prevention and health promotion?<br>ଆପଣ ହେଲ୍‌ଥ ପ୍ରମୋସନ୍ ବା ସ୍ୱାସ୍ଥ୍ୟରେ ଉନ୍ନତି ପାଇଁ ଗୋଷ୍ଠୀ କର୍ମଚାରୀମାନଙ୍କୁ ତାଲିମ୍ ଦେବା ପାଇଁ ପ୍ରଶିକ୍ଷକ ଭାବରେ ଅଂଶଗ୍ରହଣ କରିବାକୁ ଏବଂ ରୋଗ/ଅସୁସ୍ଥତା ନିରାକରଣ କରିବା ପାଇଁ ନେତୃତ୍ୱ ନେବାକୁ ଚାହିଁବେ କି?	Attitude<br>ମନୋଭାବ		
	[illegible]0.	Do you know what are the sources shared by government for health information?<br>ସରକାରଙ୍କ ସ୍ୱାସ୍ଥ୍ୟ ସୂଚନାର ଉତ୍ସ (sources) କ'ଣ ରହିଥାଏ ଆପଣ ଜାଣିଛନ୍ତି କି?	Knowledge<br>ଜ୍ଞାନ		
	[illegible]1.	Do you prefer to advise public on safe and rational use of medical products?<br>ଆପଣ ଚିକିତ୍ସାରେ ବ୍ୟବହୃତ ସାମଗ୍ରୀଗୁଡ଼ିକର ସୁରକ୍ଷିତ ଏବଂ ଯଥାର୍ଥ ବ୍ୟବହାର ବିଷୟରେ ଜନସାଧାରଣଙ୍କୁ ସଚେତନ କରିବାକୁ ଚାହିଁବେ କି?	Attitude<br>ମନୋଭାବ		
	[illegible]2.	Do you think it is worth to prepare the list of medical products and arrange their logistics required for outreach camps?<br>ଆପଣ ଭାବୁଛନ୍ତି କି ବାହ୍ୟ ସ୍ଥଳରେ ଶିବିର (outreach camps) ପାଇଁ ଆବଶ୍ୟକ ଚିକିତ୍ସାରେ ବ୍ୟବହୃତ ସାମଗ୍ରୀ ଗୁଡ଼ିକର ତାଲିକା କରିବା ଓ ଆନୁସଙ୍ଗିକ ଜିନିଷଗୁଡ଼ିକର (logistics) ବ୍ୟବସ୍ଥା କରିବା ମହତ୍ତ୍ୱପୂର୍ଣ୍ଣ ହୋଇଥାଏ?	Attitude<br>ମନୋଭାବ		
	[illegible]3.	Do you know what the role of pharmacist in the disaster management team could be?<br>ଆପଣ ବିପର୍ଯ୍ୟୟ ପରିଚାଳନା ଦଳରେ ଫାର୍ମାସିଷ୍ଟଙ୍କର ଭୂମିକା କ'ଣ ହୋଇପାରେ ଜାଣନ୍ତି କି?	Knowledge<br>ଜ୍ଞାନ		
	[illegible]4.	Do you prefer to be the part of disaster management team?<br>ଆପଣ ବିପର୍ଯ୍ୟୟ ପରିଚାଳନା ଦଳର ଅଂଶ ହେବାକୁ ପସନ୍ଦ କରିବେ କି?	Attitude<br>ମନୋଭାବ		
	[illegible]5.	Do you know the availability of government guidelines for accepting medical products donations?<br>ଆପଣ ଚିକିତ୍ସାରେ ବ୍ୟବହୃତ ସାମଗ୍ରୀ ଅନୁଦାନ (donations) ସ୍ୱୀକାର କରିବା ପାଇଁ ଉପଲବ୍ଧ ସରକାରୀ	Knowledge<br>ଜ୍ଞାନ		

		ମାର୍ଗଦର୍ଶିକା ବିଷୟରେ ଜାଣନ୍ତି କି?			
	6.	Do you know how to manage medicines during disaster situation?<br>ବିପର୍ଯ୍ୟୟ ପରିସ୍ଥିତି ସମୟରେ ଆପଣ କିପରି ଭାବରେ ଔଷଧଗୁଡ଼ିକର ପରିଚାଳନା କରିବେ ଜାଣନ୍ତି କି?	Knowledge<br>ଜ୍ଞାନ		
	7.	If require, could you prefer to provide first aid during disaster management?<br>ଯଦି ଆବଶ୍ୟକ ହୁଏ, ଆପଣ ବିପର୍ଯ୍ୟୟ ପରିଚାଳନା ସମୟରେ ପ୍ରାଥମିକ ଚିକିତ୍ସା ପ୍ରଦାନ କରିବାକୁ ଚାହିଁବେ କି?	Attitude<br>ମନୋଭାବ		
5	8.	Do you know what the laws/ acts are applied to medical products as supplied to health facilities?<br>ସ୍ୱାସ୍ଥ୍ୟ ବ୍ୟବସ୍ଥାକୁ ଯୋଗାଇ ଦିଆଯାଉଥିବା ଚିକିତ୍ସାରେ ବ୍ୟବହୃତ ସାମଗ୍ରୀଗୁଡ଼ିକ ପାଇଁ ପ୍ରଯୁଜ୍ୟ ଆଇନ /ନିୟମଗୁଡ଼ିକ ବିଷୟରେ ଆପଣ ଜାଣନ୍ତି କି?	Knowledge<br>ଜ୍ଞାନ		
	9.	Do you prefer to make an assessment of the current operational practices in regard to regulatory mechanism compliance?<br>ବର୍ତ୍ତମାନର କାର୍ଯ୍ୟଶୈଳୀରେ ଆଇନଗତ ଆବଶ୍ୟକତା ଠିକ୍ ଭାବରେ ପାଳନ କରାଯାଉଛି ,ତାହା ମୂଲ୍ୟାୟନ କରିବାକୁ ଆପଣ ଚାହିଁବେ କି?	Attitude<br>ମନୋଭାବ		
	0.	Do you know what the potential areas of improvement including the narcotics and psychotropic agents in terms of regulatory compliance could be?<br>ଆଇନଗତ ଆବଶ୍ୟକତା ଠିକ୍ ଭାବରେ ପାଳନ ଦୃଷ୍ଟିରୁ ଆଭ୍ୟାସ ଉତ୍ପନ୍ନ ହେଉଥିବା ଔଷଧ ତଥା ସାଇକୋଟ୍ରୋପିକ ଏଜେଣ୍ଟ ସହିତ ଆଉ କେଉଁ କ୍ଷେତ୍ରରେ ଉନ୍ନତି ଅଣାଯିବା ଆବଶ୍ୟକ ବୋଲି ଆପଣ ଭାବୁଛନ୍ତି?	Knowledge<br>ଜ୍ଞାନ		
	1.	Do you know the term “Potential for abuse”?<br>ଆପଣ ‘‘ଦୁରୁପଯୋଗର ସମ୍ଭାବନା’’ (“Potential for abuse”) ବିଷୟରେ ଜାଣନ୍ତି କି?	Knowledge<br>ଜ୍ଞାନ		
	2.	Do you feel worth to put effort to discourage the abuse of medical products?<br>ଚିକିତ୍ସାରେ ବ୍ୟବହୃତ ସାମଗ୍ରୀଗୁଡ଼ିକର ଦୁରୁପଯୋଗକୁ ନିରୁତ୍ସାହିତ କରିବା ପାଇଁ ଉଦ୍ୟମ କରିବା ଗୁରୁତ୍ୱପୂର୍ଣ୍ଣ ବୋଲି ଆପଣ ଅନୁଭବ କରନ୍ତି କି?	Attitude<br>ମନୋଭାବ		
	3.	Do you know about the latest state drug policy?<br>ଆପଣ ସଦ୍ୟ ରାଜ୍ୟ ଔଷଧ ନୀତି ବିଷୟରେ ଜାଣନ୍ତି କି?	Knowledge<br>ଜ୍ଞାନ		
7	4.	Do you think it is worth to follow the code of ethics of Pharmacy Council of India and as given in Pharmacy Practice Regulation?<br>ଆପଣ ଭାବୁଛନ୍ତି କି ଫାର୍ମାସି କାଉନସିଲ୍ ଅଫ୍ ଇଣ୍ଡିଆର ନୈତିକତା ଆଚରଣ ସଂହିତା ଏବଂ ଫାର୍ମାସି ପ୍ରାକ୍ଟିସ୍ ରେଗୁଲେସନ୍ ଦ୍ୱାରା ଦିଆଯାଇଥିବା ନୀତି ଅନୁସରଣ କରିବା ଉଚିତ୍?	Attitude<br>ମନୋଭାବ		
	5.	Do you think it is worth to maintain the confidentiality of patient's illness, and his/her	Attitude<br>ମନୋଭାବ		

		treatment?<br>ଆପଣ ଭାବୁଛନ୍ତି କି ରୋଗୀଙ୍କର ଅସୁସ୍ଥତା ଏବଂ ତାଙ୍କର ଚିକିତ୍ସାର ଗୋପନୀୟତା ବଜାୟ ରଖିବା ମହତ୍ୱପୂର୍ଣ୍ଣ ହୋଇଥାଏ?			
	6.	Do you know what the Pharmacist's patient care responsibilities are?<br>ଫାର୍ମାସିଷ୍ଟଙ୍କର ରୋଗୀଙ୍କ ଯତ୍ନ ଦାୟିତ୍ୱ ବିଷୟରେ ଆପଣ ଜାଣନ୍ତି କି?	Knowledge<br>ଜ୍ଞାନ		
	7.	Do you know the term "Conflict of Interest" and "Perceived conflict of Interest"?<br>ଆପଣ ''ଆପଣଙ୍କ ନିଷ୍ପତ୍ତି ବ୍ୟକ୍ତିଗତ ପସନ୍ଦ ଦ୍ୱାରା ପ୍ରଭାବିତ ହେବା'' ଏବଂ ''ପୂର୍ବରୁ ରହିଥିବା ଧାରଣା ଅନୁଯାୟୀ ନିଷ୍ପତ୍ତି ପ୍ରଭାବିତ ହେବା'' ("Conflict of Interest" and "Perceived conflict of Interest") ବିଷୟରେ ଜାଣନ୍ତି କି?	Knowledge<br>ଜ୍ଞାନ		
	8.	Are you willing to disclose the conflict of interest?<br>ଆପଣ ''ଆପଣଙ୍କ ନିଷ୍ପତ୍ତି ବ୍ୟକ୍ତିଗତ ପସନ୍ଦ ଦ୍ୱାରା ପ୍ରଭାବିତ ହେବା'' ("Conflict of Interest") ବିଷୟରେ ପ୍ରକାଶ କରିବାକୁ ଇଚ୍ଛୁକ କି?	Attitude<br>ମନୋଭାବ		
	9.	Do you think the good practices could be helpful in minimizing/ avoiding the conflict of Interest?<br>ଆପଣ ଭାବୁଛନ୍ତି କି ଉତ୍ତମ ବିଧି ''ଆପଣଙ୍କ ନିଷ୍ପତ୍ତି ବ୍ୟକ୍ତିଗତ ପସନ୍ଦ ଦ୍ୱାରା ପ୍ରଭାବିତ ହେବା'' ("Conflict of Interest") ସର୍ବନିମ୍ନ କରିବା ପାଇଁ/ ଏଡ଼ାଇବା ପାଇଁ ସହାୟକ ହୋଇପାରେ ?	Attitude<br>ମନୋଭାବ		
9	0.	Do you prefer to maintain professional relationship with other primary health care team members?<br>ଆପଣ ପ୍ରାଥମିକ ସ୍ୱାସ୍ଥ୍ୟ ଯତ୍ନ ଦଳର ଅନ୍ୟ ସଦସ୍ୟମାନଙ୍କ ସହ ପେଶାଦାର ସମ୍ପର୍କ ବଜାୟ ରଖିବାକୁ ଚାହାନ୍ତି କି?	Attitude<br>ମନୋଭାବ		
	1.	Do you prefer to participate as a team member in delivering health services?<br>ଆପଣ ସ୍ୱାସ୍ଥ୍ୟ ସେବା ପ୍ରଦାନ କରିବା ପାଇଁ ଦଳର ଜଣେ ସଦସ୍ୟ ଭାବରେ କାମ କରିବାକୁ ଚାହାନ୍ତି କି?	Attitude<br>ମନୋଭାବ		
10	2.	Do you know the government of Odisha's order for dispensing medicines for specific conditions in absence of medical officer?<br>ଆପଣ ଓଡ଼ିଶା ସରକାରଙ୍କ ନିୟମ ଅନୁଯାୟୀ ଡାକ୍ତର ନଥିବା ସମୟରେ ଫାର୍ମାସିଷ୍ଟ ରୋଗୀଙ୍କୁ କେଉଁ ଔଷଧ ସେବନ କରିବାକୁ ପରାମର୍ଶ ଦେଇପାରିବେ ଜାଣନ୍ତି କି?	Knowledge<br>ଜ୍ଞାନ		
	3.	Do you know what those conditions for which government of Odisha permits to dispense medicines in absence of medical officer (without medical officer's prescription)?<br>ଓଡ଼ିଶା ସରକାରଙ୍କ ନିୟମ ଅନୁଯାୟୀ ଡାକ୍ତର ନଥିବା ସମୟରେ ଜଣେ ଫାର୍ମାସିଷ୍ଟ କେଉଁ କେଉଁ ରୋଗ ପାଇଁ ଔଷଧ ପରାମର୍ଶ ଦେଇପାରିବେ ଜାଣନ୍ତି କି?	Knowledge<br>ଜ୍ଞାନ		

	4.	Do you know the list of medicines for which government of Odisha has empowered Pharmacist to dispense in absence of medical officer (without medical officer's prescription)?<br>ଓଡ଼ିଶା ସରକାରଙ୍କ ନିୟମ ଅନୁଯାୟୀ ଡାକ୍ତର ନଥିବା ସମୟରେ ଜଣେ ଫାର୍ମାସିଷ୍ଟ ରୋଗୀଙ୍କୁ ଯେଉଁ ଔଷଧ ସେବନ କରିବାକୁ ପରାମର୍ଶ ଦେଇପାରିବେ ତାହାର ତାଲିକା ବିଷୟରେ ଜାଣନ୍ତି କି?	Knowledge<br>ଜ୍ଞାନ		
	5.	Do you know how to diagnose (Diagnostic tests/ Sign and Symptoms) the cases of Malaria, Upper Respiratory Tract Infection, Scabies?<br>ଆପଣ କିପରି ଭାବରେ ମ୍ୟାଲେରିଆ, ଉପର ଶ୍ୱାସମାର୍ଗ ସଂକ୍ରମଣ, ଯାଦୁ ବା ଚର୍ମ ରୋଗ ଚିହ୍ନିବେ(ନୈଦାନିକ ପରୀକ୍ଷାଗୁଡ଼ିକ/ଲକ୍ଷଣ ଏବଂ ସଙ୍କେତ) ଜାଣନ୍ତି କି?	Knowledge<br>ଜ୍ଞାନ		
	6.	Do you know how to select the medicine for a condition from permissible medicine list, for example- selection of Antibiotic for fever- Azithromycin/ Cefixime / Amoxycillin ± Clavulanic Acid. ?<br>ଆପଣ ପରାମର୍ଶ ଦେବା ପାଇଁ ଅନୁମତିଯୋଗ୍ୟ ଔଷଧ ତାଲିକାରୁ ଏକ ରୋଗ ପାଇଁ ଔଷଧ କିପରି ବାଛିବେ ଜାଣନ୍ତି କି, ଉଦାହରଣ ସ୍ୱରୂପ ଜ୍ୱର ପାଇଁ ଏକ ଆଣ୍ଟିବାୟୋଟିକ୍ ବାଛିବା - ଆଜିଥ୍ରୋମାଇସିନ୍ / ସେଫିକ୍ସିନ୍ / ଆମୋକ୍ସିସିଲିନ୍ ± କ୍ଲାଭୁଲାନିକ୍ ଏସିଡ୍ (Azithromycin/ Cefixime / Amoxycillin ± Clavulanic Acid)?	Knowledge<br>ଜ୍ଞାନ		
	7.	Do you know when the patients are supposed to be referred to the higher health facilities?<br>ରୋଗୀଙ୍କୁ କେତେବେଳେ ଅଧିକ ଉନ୍ନତ ସ୍ୱାସ୍ଥ୍ୟ ବ୍ୟବସ୍ଥାକୁ ରେଫର୍ କରାଯିବ ଆପଣ ଜାଣନ୍ତି କି?	Knowledge<br>ଜ୍ଞାନ		
	8.	Do you prefer to refer the patient to the higher facilities, if required (In absence of medical officer)?<br>ଯଦି ଆବଶ୍ୟକ ହୁଏ (ଡାକ୍ତରଙ୍କ ଅନୁପସ୍ଥିତିରେ) ଆପଣ ରୋଗୀଙ୍କୁ ଉନ୍ନତ ବ୍ୟବସ୍ଥାକୁ ରେଫର୍ କରିବାକୁ ଚାହିଁବେ କି?	Attitude<br>ମନୋଭାବ		
11	9.	Do you know what Continuing Professional Education/ Development programmes are available for pharmacists?<br>ଆପଣ ଫାର୍ମାସିଷ୍ଟଙ୍କ ପାଇଁ କି କି ବୃତ୍ତିଗତ ଶିକ୍ଷା/ବିକାଶମୂଳକ କାର୍ଯ୍ୟକ୍ରମ ଚାଲୁ ରହିଛି ଜାଣନ୍ତି କି?	Knowledge<br>ଜ୍ଞାନ		
	0.	If programs are available, would you prefer to attend those Continuing Professional Education/ Development programmes?<br>ଯଦି ପ୍ରୋଗ୍ରାମଗୁଡ଼ିକ ଉପଲବ୍ଧ ରହିଥାଏ ଆପଣ ସେହି ଚାଲୁ ରହିଥିବା ବୃତ୍ତିଗତ ଶିକ୍ଷା/ବିକାଶମୂଳକ କାର୍ଯ୍ୟକ୍ରମରେ ଯୋଗ ଦେବାକୁ ଚାହିଁବେ କି?	Attitude<br>ମନୋଭାବ		
	1.	Do you know the governments training programs for pharmacists?<br>ଆପଣ ଫାର୍ମାସିଷ୍ଟଙ୍କ ପାଇଁ ସରକାରଙ୍କର ତାଲିମ୍	Knowledge<br>ଜ୍ଞାନ		

		କାର୍ଯ୍ୟକ୍ରମ ବିଷୟରେ ଜାଣନ୍ତି କି?			
	2.	Do you prefer to attend government organized programs for pharmacists?<br>ଆପଣ ଫାର୍ମାସିଷ୍ଟମାନଙ୍କ ପାଇଁ ସରକାରଙ୍କ ଦ୍ୱାରା ଆୟୋଜିତ କାର୍ଯ୍ୟକ୍ରମଗୁଡ଼ିକରେ ଯୋଗ ଦେବାକୁ ଚାହାନ୍ତି କି?	Attitude<br>ମନୋଭାବ		

C.2. Observational Tools (Skills)

C.2. ଅନୁଧାନମୂଳକ ସାଧନଗୁଡ଼ିକ (ଦକ୍ଷତା)

Domain Code<br>ଡୋମେନ ବା କ୍ଷେତ୍ର କୋଡ୍	S. N.<br>କ୍ର.ସଂ.	Observation Points<br>ଅନୁଧାନ କରିବାର ବିଷୟଗୁଡ଼ିକ	Means of Verification<br>ଯାଞ୍ଚର ମାଧ୍ୟମ	Response (Score)<br>ଜବାବ (ସ୍କୋର୍)
1		Realignment of Infrastructure<br>ରହିଥିବା ଭୌତିକ ବ୍ୟବସ୍ଥାରେ (Infrastructure) ପରିବର୍ତ୍ତନ ବା ଉନ୍ନତିକରଣ	Identification of the areas of realignment<br>କେଉଁ କ୍ଷେତ୍ରରେ ପରିବର୍ତ୍ତନ ଦରକାର ଚିହ୍ନଟ କରିବା<br>Physical verification of the feasibility of realignment<br>କିପରି ଭାବରେ ଏହି ପରିବର୍ତ୍ତନକୁ ଲାଗୁ କରିବେ ଯାଞ୍ଚ କରି ଦେଖିବା	
		Labelling of Places of segregation in the Pharmacy and Pharmacy Store<br>ଫାର୍ମାସି ଏବଂ ଫାର୍ମାସି ଷ୍ଟୋରରେ ପୃଥକୀକରଣ ପାଇଁ ସ୍ଥାନଗୁଡ଼ିକର ଲେବଲିଂ ବା ନାମ ଦ୍ୱାରା ଚିହ୍ନିତ କରିବା	Places labelled or not<br>ସ୍ଥାନଗୁଡ଼ିକ ଲେବଲ୍ କରାଯାଇଛି ବା କରାଯାଇନାହିଁ<br>Quality of label whether the label mark is permanent<br>ଲେବଲ୍‌ର ଗୁଣବତ୍ତା ଏହି ଲେବଲ୍‌ର ମାର୍କ ଚିରସ୍ଥାୟୀ କି	
		Identification of Storage area in storeroom including shelves (Objective is to maintain potency and easy accessibility)<br>ଷ୍ଟୋର ରୁମ୍‌ରେ ଷ୍ଟୋରେଜ୍ କ୍ଷେତ୍ର ସହିତ ସେଲ୍‌ଫଗୁଡ଼ିକର ଚିହ୍ନଟକରଣ (ଉଦ୍ଦେଶ୍ୟ ହେଉଛି ଏହାର କାର୍ଯ୍ୟକ୍ଷମତା ବଜାୟ ରଖିବା ଓ ସହଜରେ ପାଇବା)	Whether the storage area is identified for the temperature sensitive and moisture sensitive medical products.<br>ତାପମାତ୍ରା ପ୍ରତି ସମ୍ବେଦନଶୀଳ ଏବଂ ଆର୍ଦ୍ରତା ପ୍ରତି ସମ୍ବେଦନଶୀଳ ଚିକିତ୍ସାରେ ବ୍ୟବହୃତ ସାମଗ୍ରୀ ପାଇଁ ସଂରକ୍ଷଣ କ୍ଷେତ୍ର ଚିହ୍ନିତ କରାଯାଇଛି କି□	
		Organizing the Medical Products in Pharmacy and Pharmacy store<br>ଫାର୍ମାସି ଏବଂ ଫାର୍ମାସି ଷ୍ଟୋରରେ ଚିକିତ୍ସାରେ ବ୍ୟବହୃତ ସାମଗ୍ରୀଗୁଡ଼ିକ ସାଇତି ରଖିବା	Whether the storage place is labelled with type /Name<br>ରଖିବାର ସ୍ଥାନକୁ ପ୍ରକାର/ନାମ ସହିତ ଲେବଲ୍ କରାଯାଇଛି କି	
		Maintenance of Pest control record<br>କୀଟପତଙ୍ଗ ନିୟନ୍ତ୍ରଣ (Pest control) ପଦ୍ଧତି ସଠିକ ଭାବରେ ରେକର୍ଡ କରିବା	The display of pest control record with essential detail<br>ଆବଶ୍ୟକ ବିବରଣୀ ସହ କୀଟପତଙ୍ଗ ନିୟନ୍ତ୍ରଣ (Pest control) ପଦ୍ଧତି ରେକର୍ଡ ଦେଖାଇବା	
		Storage of Medical Products in	Identification of Medical Products	

		safe and secure manner<br>ଚିକିତ୍ସାରେ ବ୍ୟବହୃତ ସାମଗ୍ରୀଗୁଡ଼ିକର ନିରାପଦ ଏବଂ ସୁରକ୍ଷିତ ଭାବରେ ସଂରକ୍ଷଣ	susceptible to misuse and abuse<br>ଅପବ୍ୟବହାର ଏବଂ ଦୁରୁପଯୋଗର ସଂଭାବନା ରହିଥିବା ଚିକିତ୍ସାରେ ବ୍ୟବହୃତ ସାମଗ୍ରୀଗୁଡ଼ିକ ଚିହ୍ନଟ କରିବା<br>Medical products kept under lock and key (Specially for habit forming medical products)<br>ଚିକିତ୍ସାରେ ବ୍ୟବହୃତ ସାମଗ୍ରୀଗୁଡ଼ିକ ତାଲା ଚାବି ପକାଇ ରଖାଯାଇଥାଏ (ବିଶେଷକରି ନିଶା ଅଭ୍ୟାସ ସୃଷ୍ଟି କରୁଥିବା ଚିକିତ୍ସାରେ ବ୍ୟବହୃତ ସାମଗ୍ରୀଗୁଡ଼ିକ)	
		Filling up the Indenting Form<br>ଇଣ୍ଡେଣ୍ଟ କରିବା ପାଇଁ ଫର୍ମ ପୂରଣ କରିବା	Indenting Form Parameters<br>ଇଣ୍ଡେଣ୍ଟ କରିବା ଫର୍ମର ମାନଦଣ୍ଡଗୁଡ଼ିକ<br>Strength, type and Quantity of Medical Products<br>ଚିକିତ୍ସାରେ ବ୍ୟବହୃତ ସାମଗ୍ରୀ ଗୁଡ଼ିକର କ୍ଷମତା, ପ୍ରକାର ଏବଂ ପରିମାଣ	
		Use E-NIRAMAYA-<br>ଇ-ନିରାମୟ ବ୍ୟବହାର କରନ୍ତୁ	Data entry -Inputs Up to date<br>ଡାଟା ଏଣ୍ଟ୍ରି -ଇନପୁଟ୍ ଅପଟୁଡେଟ୍ ବା ଅଦ୍ୟତନ କରନ୍ତୁ<br>Generate Monthly Report<br>ମାସିକ ରିପୋର୍ଟ ଉତ୍ପନ୍ନ କରନ୍ତୁ	
		Identify area of Improvement and tracking the progress based on E-NIRAMAYA-<br>ଇ-ନିରାମୟ ଆଧାରରେ କାର୍ଯ୍ୟରେ ସୁଧାର ପାଇଁ କ୍ଷେତ୍ରଗୁଡ଼ିକ ଚିହ୍ନଟ କରିବା ଏବଂ ଅଗ୍ରଗତି ଅନୁଧ୍ୟାନ କରିବା	Listing of the areas require improvement<br>କାର୍ଯ୍ୟରେ ସୁଧାର ଆବଶ୍ୟକ ହେଉଥିବା କ୍ଷେତ୍ରଗୁଡ଼ିକର ତାଲିକା କରିବା<br>Sharing those areas requiring improvement with other team members<br>କେଉଁ କେଉଁ କ୍ଷେତ୍ରରେ ସୁଧାର ଆଣିବା ଆବଶ୍ୟକ, ଦଳର ଅନ୍ୟ ସଦସ୍ୟମାନଙ୍କୁ ଜଣାଇବା<br><br>Tracking the action taken on areas require Improvement<br>ସୁଧାର ଆଣିବା ଆବଶ୍ୟକ ହେଉଥିବା କ୍ଷେତ୍ରଗୁଡ଼ିକରେ ଯେଉଁ କାର୍ଯ୍ୟାନୁଷ୍ଠାନ ଗ୍ରହଣ କରାଯାଏ ସେସବୁ ଟ୍ରାକ୍ କରିବା	
		Condemnation Policy / Condemnation Committee functionality<br>କଣ୍ଡେମନେସନ୍ ନୀତି/କଣ୍ଡେମନେସନ୍ କମିଟିର କାର୍ଯ୍ୟକାରିତା	Policy document<br>ନୀତି ଦଲିଲ<br>Committee Constitution document<br>କମିଟି ଗଠନ ଦଲିଲ୍<br>Last Committee meeting minutes<br>ଗତ କମିଟି ବୈଠକର ସାରାଂଶ	
		Register maintenance -Pharmacy<br>ରେଜିଷ୍ଟର୍ ରକ୍ଷଣାବେକ୍ଷଣ -ଫାର୍ମାସି	Register availability for Medical Products stock<br>ଚିକିତ୍ସାରେ ବ୍ୟବହୃତ ସାମଗ୍ରୀ ପାଇଁ ଷ୍ଟକ୍ ରେଜିଷ୍ଟର (ବହି) ଅଛି କି ନାହିଁ<br>Correctness of Entries<br>ସଠିକ୍ ଭାବରେ ବିବରଣୀ ଲେଖିବା<br>Completeness of entries<br>ସମ୍ପୂର୍ଣ୍ଣ ଭାବରେ ବିବରଣୀ ଲେଖିବା	

		Tracking of Government notifications on frozen or NSQ medical products<br>ଅଚଳନ୍ତି ଘୋଷଣା କରାଯାଇଥିବା କିମ୍ବା ବ୍ୟବହାର ପାଇଁ ଅନୁପଯୋଗୀ (frozen or NSQ) ଚିକିତ୍ସାରେ ବ୍ୟବହୃତ ସାମଗ୍ରୀ ସମ୍ପର୍କିତ ସରକାରୀ ନିର୍ଦ୍ଦେଶନାମା	Availability of Records<br>ରେକର୍ଡଗୁଡ଼ିକର ଉପଲବ୍ଧତା<br>Recent State Government notification available<br>ବର୍ତ୍ତମାନର ରାଜ୍ୟ ସରକାରଙ୍କର ବିଜ୍ଞପ୍ତି ଉପଲବ୍ଧ ରହିଛି<br>Central Drug Standard Control Organization (CDSCO) notification<br>ସେଣ୍ଟ୍ରାଲ୍ ଡ୍ରଗ୍‌ଷ୍ଟାଣ୍ଡାର୍ଡ କଣ୍ଟ୍ରୋଲ୍ ଅର୍ଗାନାଇଜେସନ୍ (ସିଡିଏସସିଓ) ବିଜ୍ଞପ୍ତି	
		Identification of frozen or NSQ Medical Products<br>ଅଚଳନ୍ତି ଘୋଷଣା କରାଯାଇଥିବା କିମ୍ବା ବ୍ୟବହାର ପାଇଁ ଅନୁପଯୋଗୀ (frozen or NSQ) ଚିକିତ୍ସାରେ ବ୍ୟବହୃତ ସାମଗ୍ରୀଗୁଡ଼ିକ ଚିହ୍ନଟ କରିବା	Matching the medical products with product name and batch number of Government notification<br>ଚିକିତ୍ସାରେ ବ୍ୟବହୃତ ସାମଗ୍ରୀଗୁଡ଼ିକର ନାମ ଏବଂ ସରକାରୀ ନିର୍ଦ୍ଦେଶନାମା ଅନୁଯାୟୀ ବ୍ୟାଚ୍ ନମ୍ବରରେ ରହିଥିବା ନାମପତ୍ର (Label) ସମାନ ଅଛି କି ନା ଯାଞ୍ଚ କରିବା	
		Mechanism for retrieving frozen or NSQ medical products<br>ଅଚଳନ୍ତି ଘୋଷଣା କରାଯାଇଥିବା କିମ୍ବା ବ୍ୟବହାର ପାଇଁ ଅନୁପଯୋଗୀ (frozen or NSQ) ଚିକିତ୍ସାରେ ବ୍ୟବହୃତ ସାମଗ୍ରୀଗୁଡ଼ିକ ଫେରସ୍ତ ଆଣିବା ପାଇଁ ବ୍ୟବସ୍ଥା	Communication with the department like Labour room, emergency etc<br>ପ୍ରସୂତି କକ୍ଷ, ଆଶୁ ଚିକିତ୍ସା ବିଭାଗ (Labour room, emergency) ଇତ୍ୟାଦି ସହିତ ଯୋଗାଯୋଗ କରିବା<br>Medical products retrieval documentation<br>ଚିକିତ୍ସାରେ ବ୍ୟବହୃତ ସାମଗ୍ରୀଗୁଡ଼ିକ ଫେରସ୍ତ ଆଣିବାର ବିବରଣୀ ଲେଖିବା	
		Segregation of frozen or NSQ Medical Products<br>ଚିକିତ୍ସାରେ ବ୍ୟବହୃତ ଅଚଳନ୍ତି ଘୋଷଣା କରାଯାଇଥିବା କିମ୍ବା ବ୍ୟବହାର ପାଇଁ ଅନୁପଯୋଗୀ (frozen or NSQ) ସାମଗ୍ରୀଗୁଡ଼ିକ ଅଲଗା କରି ରଖିବା	Availability of Identified area for separating for frozen or NSQ Medical Products<br>ଚିକିତ୍ସାରେ ବ୍ୟବହୃତ ଅଚଳନ୍ତି ଘୋଷଣା କରାଯାଇଥିବା କିମ୍ବା ବ୍ୟବହାର ପାଇଁ ଅନୁପଯୋଗୀ (frozen or NSQ) ସାମଗ୍ରୀଗୁଡ଼ିକ ଅଲଗା ରଖିବା ପାଇଁ କ୍ଷେତ୍ର ଚିହ୍ନଟ କରାଯାଇଛି<br>Frozen or NSQ medical products reflected in the store/ stock register<br>ଷ୍ଟୋର/ଷ୍ଟକ୍ ରେଜିଷ୍ଟରରେ ଚିକିତ୍ସାରେ ବ୍ୟବହୃତ ଅଚଳନ୍ତି ଘୋଷଣା କରାଯାଇଥିବା କିମ୍ବା ବ୍ୟବହାର ପାଇଁ ଅନୁପଯୋଗୀ (frozen or NSQ) ସାମଗ୍ରୀଗୁଡ଼ିକ ଲେଖା ଅଛି କି ନାହିଁ	
3		Ensuring the right medical products for dispensing to the patient<br>ରୋଗୀଙ୍କୁ ବିତରଣ କରିବା ପାଇଁ ଚିକିତ୍ସାରେ ବ୍ୟବହୃତ ସଠିକ୍ ସାମଗ୍ରୀଗୁଡ଼ିକ ସୁନିଶ୍ଚିତ କରିବା	Verification at two level-<br>ଦୁଇଟି ସ୍ତରରେ ଯାଞ୍ଚକରଣ -<br>During prescription reading<br>ଡାକ୍ତରୀ ଚିଠା (prescription) ପଢ଼ିବା ସମୟରେ<br>During the dispensing<br>ବିତରଣ କରିବା ସମୟରେ	
		Adequate auxiliary labelling of medical products before dispensing to the patient<br>ରୋଗୀଙ୍କୁ ବିତରଣ କରିବା ପାଇଁ ପୂର୍ବରୁ ଚିକିତ୍ସାରେ ବ୍ୟବହୃତ ସାମଗ୍ରୀଗୁଡ଼ିକର ଯଥାର୍ଥ ଅକଜିଲାରୀ ଲେବଲିଂ କରିବା	Materials used for additional -labelling- sticker, marker etc.<br>ଅତିରିକ୍ତ - ଲେବଲିଂ ପାଇଁ ଷ୍ଟିକର୍, ମାର୍କର ଇତ୍ୟାଦି ଜିନିଷ ବ୍ୟବହାର କରାଯାଇଥାଏ<br><br>Patient name Dosage, Shake well before	

			use, For external use, etc.<br>ରୋଗୀଙ୍କର ନାମ, ପାନ ବା ମାତ୍ରା, ବ୍ୟବହାର କରିବା ପୂର୍ବରୁ ଭଲ ଭାବରେ ହଲାନ୍ତୁ, କେବଳ ବାହ୍ୟ ବ୍ୟବହାର ପାଇଁ ଇତ୍ୟାଦି	
		Medication Counselling<br>ଔଷଧ ସେବନ ପାଇଁ ପରାମର୍ଶ	Storage, dosage, frequency, timing, way/method of usage/administration, route of administration, drug interaction/ incompatibility, adverse drug reaction, diet and lifestyle modifications<br>ଘରେ କିପରି ସାଇତି ରଖିବେ, କେତେ ମାତ୍ରାରେ, କେତେ ବ୍ୟବଧାନରେ, କେଉଁ ସମୟରେ ବ୍ୟବହାର କରିବେ, ବ୍ୟବହାର / ପ୍ରୟୋଗର ପଦ୍ଧତି/ଉପାୟ, ପ୍ରୟୋଗର ମାଧ୍ୟମ, ଔଷଧଗୁଡ଼ିକର ପରସ୍ପର ସହ ପ୍ରତିକ୍ରିୟା / କେଉଁ ଔଷଧ ସହ ଏକାଠି ସେବନ କରିପାରିବେ ନାହିଁ, ଔଷଧର ଅପ୍ରତ୍ୟାଶିତ ପ୍ରଭାବଗୁଡ଼ିକ (adverse drug reaction), ଆହାର ସମ୍ପର୍କିତ କଟକଣା ଏବଂ ଜୀବନଶୈଳୀରେ ପରିବର୍ତ୍ତନ	
		Patient Queries<br>ରୋଗୀଙ୍କର ପ୍ରଶ୍ନଗୁଡ଼ିକ	Listening calmly<br>ଧ୍ୟାନ ଦେଇ ଶୁଣିବା<br>Respond in language understandable to the patient<br>ରୋଗୀ ବୁଝିପାରିବା ଭଳି ଭାଷାରେ ଉତ୍ତର ଦେବା<br>Information-Correct, Complete,<br>ସୂଚନା - ସଠିକ୍, ସମ୍ପୂର୍ଣ୍ଣ,<br>Keep it short and simple (KISS)<br>ଏହାକୁ ସଂକ୍ଷିପ୍ତ ଓ ସରଳ ରଖନ୍ତୁ (କେଆଇଏସଏସ)	
		Mechanism to return unused, unwanted, or expired medical products to Pharmacy<br>ଅବ୍ୟବହୃତ, ଅବାଞ୍ଛିତ କିମ୍ୱା ମିଆଦ ଶେଷ ହୋଇଥିବା ଚିକିତ୍ସାରେ ବ୍ୟବହୃତ ସାମଗ୍ରୀଗୁଡ଼ିକ ଫାର୍ମାସିକୁ ଫେରସ୍ତ କରିବା ପାଇଁ ବ୍ୟବସ୍ଥା	Information shared during dispensing<br>ବିତରଣ କରିବା ସମୟରେ କି କି ସୂଚନା ଦିଆଯାଇଥାଏ<br>Recording of returned medical products<br>ଫେରସ୍ତ ଆସିଥିବା ଚିକିତ୍ସାରେ ବ୍ୟବହୃତ ସାମଗ୍ରୀଗୁଡ଼ିକର ରେକର୍ଡ କରିବା	
8		Communication<br>ଯୋଗାଯୋଗ	Active listening<br>ଧ୍ୟାନପୂର୍ବକ ଶୁଣିବା<br>Verbal Communication-Clear, Precise<br>ମୌଖିକ ଯୋଗାଯୋଗ - ସ୍ପଷ୍ଟ, ସଂକ୍ଷିପ୍ତ<br>Non-verbal communication<br>ଅଣ-ମୌଖିକ ଯୋଗାଯୋଗ<br>Written Communication-Check documents like – Leave application; Indenting to Procure required Items etc.<br>ଲିଖିତ ଯୋଗାଯୋଗ ଦଲିଲଗୁଡ଼ିକ ଯାଞ୍ଚ କରନ୍ତୁ - ଯେପରିକି ଛୁଟି ପାଇଁ ଆବେଦନପତ୍ର ; ଆବଶ୍ୟକ ସାମଗ୍ରୀ ଆହରଣ ପାଇଁ ଇଣ୍ଡେଣ୍ଟ କରିବା ଇତ୍ୟାଦି<br>Use of terms understandable to the patient<br>ରୋଗୀ ବୁଝିପାରୁଥିବା ପରି ଶବ୍ଦର ବ୍ୟବହାର<br>Cultural awareness and sensitivity	

			ସାଂସ୍କୃତିକ ସଚେତନତା ଏବଂ ସମ୍ବେଦନଶୀଳତା<br>Responsiveness towards Patient queries including medical products<br>ରୋଗୀଙ୍କର ଔଷଧ ସମ୍ପର୍କିତ ପ୍ରଶ୍ନ ସହ ସମସ୍ତ ପ୍ରଶ୍ନର ତତ୍ପରତାର ସହ ଉତ୍ତର ଦେବା	
		Effective interdisciplinary/intra professional Communication<br>ବିଭିନ୍ନ ବିଭାଗ/ବିଭିନ୍ନ ବୃତ୍ତି ମଧ୍ୟରେ ପ୍ରଭାବଶାଳୀ ଯୋଗାଯୋଗ	Observe his/her interdisciplinary/intra professional Communication.<br>ବିଭିନ୍ନ ବିଭାଗ/ବିଭିନ୍ନ ବୃତ୍ତି ମଧ୍ୟରେ ତାଙ୍କର ଯୋଗାଯୋଗ ଅନୁଧ୍ୟାନ କରନ୍ତୁ□<br>Whether he fulfils his role in the medical team<br>ସେ ଡାକ୍ତରୀ ଦଳରେ ତାଙ୍କର ଦାୟିତ୍ୱ ପୂରଣ କରନ୍ତି କି	
		Official Communication<br>ଔପଚାରିକ ଯୋଗାଯୋଗ	Content should be<br>• Complete,<br>• Clear<br>• Precise<br><br>ବିଷୟବସ୍ତୁ<br><br>• ସମ୍ପୂର୍ଣ୍ଣ<br>• ସ୍ପଷ୍ଟ<br>• ସଂକ୍ଷିପ୍ତ<br>ହେବା ଉଚିତ	

C.3. Mini Clinical Evaluation of selected Conditions (Skills)

C.3. ଚୟନ କରାଯାଇଥିବା ଅବସ୍ଥାଗୁଡ଼ିକର ସଂକ୍ଷିପ୍ତ ନୈଦାନିକ ମୂଲ୍ୟାୟନ (ଦକ୍ଷତାଗୁଡ଼ିକ)

Domain Code<br>ଡୋମେନ ବା କ୍ଷେତ୍ର କୋଡ୍	S.N.<br>କ୍ର.ସଂ.	Evaluation Points<br>ମୂଲ୍ୟାୟନର ବିଷୟଗୁଡ଼ିକ	Means of Verification<br>ଯାଞ୍ଚର ମାଧ୍ୟମ	Response (Score)<br>ଜବାବ (ସ୍କୋର୍)	Remarks<br>ଟିପ୍ପଣୀ
1		Inventory management Technique (VED)<br>ଇନଭେଣ୍ଟୋରୀ ପରିଚାଳନା କୌଶଳ (ଭିଇଡି)<br>(Inventory management Technique (VED))	Verification of medicines given<br>ଦିଆଯାଇଥିବା ଔଷଧର ଯାଞ୍ଚକରଣ<br>Grouping them into three categories<br>ସେଗୁଡ଼ିକୁ ୩ଟି ବର୍ଗରେ ବର୍ଗୀକୃତ କରିବା<br>Action taken<br>କି କାର୍ଯ୍ୟାନୁଷ୍ଠାନ ଗ୍ରହଣ କରାଯାଇଛି		
		Appropriately Organizing the medical products according to the storage need	Matching with the product and recommended storage condition.<br>ଚିକିତ୍ସାରେ ବ୍ୟବହୃତ ସାମଗ୍ରୀଗୁଡ଼ିକ		

		ସଂରକ୍ଷଣ ଆବଶ୍ୟକତା ଅନୁଯାୟୀ ଚିକିତ୍ସାରେ ବ୍ୟବହୃତ ସାମଗ୍ରୀଗୁଡ଼ିକ ଯଥାର୍ଥ ଭାବରେ ସଂଯୋଜିତ କରିବା	ଅନୁଯାୟୀ ଅନୁମୋଦିତ ସଂରକ୍ଷଣ ଅବସ୍ଥା ଯେପରିକି ତାପମାତ୍ରା ଠିକ୍ ରହିଛି କି ଯାଞ୍ଚ କରି ଦେଖିବା		
		Medical Products Identification based on epidemiological need of Catchment Population<br>ଡାକ୍ତରଖାନା ସେବା ପ୍ରଦାନ କରାଯାଉଥିବା କ୍ଷେତ୍ରର ଲୋକମାନଙ୍କ ମଧ୍ୟରେ ବହୁଜନ ବ୍ୟାପୀ ବ୍ୟାଧି ଓ ତାହାର ନିୟନ୍ତ୍ରଣ ବିଦ୍ୟା ଆବଶ୍ୟକତା ଭିତ୍ତିରେ ଚିକିତ୍ସାରେ ବ୍ୟବହୃତ ସାମଗ୍ରୀଗୁଡ଼ିକ ର ଚିହ୍ନଟକରଣ	Identification of the disease<br>ରୋଗର ଚିହ୍ନଟକରଣ<br><br>Understanding the Standard Treatment Guideline/ workflows for the identified disease.<br><br>ଚିହ୍ନିତ ରୋଗ ପାଇଁ ମାନକ ଚିକିତ୍ସା ମାର୍ଗଦର୍ଶିକା/କାର୍ଯ୍ୟପ୍ରବାହକୁ ବୁଝିବା		
		Forecasting the requirements of Medical Products- Based on the past consumption<br>ଅତୀତର ବ୍ୟବହାର ଆଧାରରେ ଚିକିତ୍ସାରେ ବ୍ୟବହୃତ ସାମଗ୍ରୀ ଗୁଡ଼ିକର ଆବଶ୍ୟକତା ପୂର୍ବାନୁମାନ କରିବା	Past Consumption Data taking consideration of stock out period<br>ଷ୍ଟକଆଉଟ୍ ବା ଷ୍ଟକ ସରିଯିବା ଅବଧିକୁ ହିସାବକୁ ନେଇ ଅତୀତରେ କେତେ ବ୍ୟବହାର ହୋଇଛି ସେହି ସୂଚନା<br>Calculation for the requirement of medical Products taking account of population growth or epidemiological transition for two years.<br>2 ବର୍ଷ ମଧ୍ୟରେ ଜନସଂଖ୍ୟା ବୃଦ୍ଧି ଏବଂ ରୋଗ ବ୍ୟାପିବାରେ ପରିବର୍ତ୍ତନକୁ ଦୃଷ୍ଟିରେ ରଖି ଚିକିତ୍ସାରେ ବ୍ୟବହୃତ ସାମଗ୍ରୀ ର ଆବଶ୍ୟକତା ହିସାବ କରିବା		
		Local Purchasing Documentation<br>ସ୍ଥାନୀୟ କ୍ରୟକୁ ନଥିଭୁକ୍ତ କରିବା	Able to fill the Standard Formats<br>ମାନକ ଫର୍ମାଟଗୁଡ଼ିକ ପୂରଣ କରିବା ପାଇଁ ସକ୍ଷମ<br>Preservation of Purchasing documents<br>କ୍ରୟ ଦଲିଲଗୁଡ଼ିକ ସଂରକ୍ଷିତ ଭାବରେ ରଖିବା<br>Any additional documentation required<br>ଅତିରିକ୍ତ କାଗଜପତ୍ର କାମ ଯଦି ଆବଶ୍ୟକ ହୁଏ		

		Contingency Plan for Stock out<br>ଷ୍ଟକ୍ ଶେଷ ହେବା ଏଡ଼ାଇବା ପାଇଁ କଣ୍ଟିଜେନ୍ସି ବା ଆକସ୍ମିକ ଆବଶ୍ୟକତା ପୂରଣ ପାଇଁ ଯୋଜନା	Local Purchasing procedures in Place<br>ସ୍ଥାନୀୟ କ୍ରୟ ପାଇଁ ପଦ୍ଧତି ରହିଛି<br>Facilitate the supplies from neighboring institutions or district store.<br>ନିକଟବର୍ତ୍ତୀ ପ୍ରତିଷ୍ଠାନ କିମ୍ବା ଜିଲ୍ଲା ଷ୍ଟୋରରୁ ଯୋଗାଣ ବ୍ୟବସ୍ଥା କରିବା		
		Verification Checklist for receiving supplies<br>ଯୋଗାଣ ଗ୍ରହଣ କରିବା ପାଇଁ ଯାଞ୍ଚ ତାଲିକା ପରଖି ଦେଖିବା	Is the pharmacist able to prepare a checklist for receiving the supplied identified medical products?<br>ଫାର୍ମାସିଷ୍ଟ ଯୋଗାଣ କରାଯାଇଥିବା ଚିକିତ୍ସାରେ ବ୍ୟବହୃତ ସାମଗ୍ରୀଗୁଡ଼ିକ ଗ୍ରହଣ କରିବା ପାଇଁ ଏକ ଯାଞ୍ଚ ତାଲିକା ପ୍ରସ୍ତୁତ କରିବାକୁ ସକ୍ଷମ ହୋଇଛନ୍ତି କି?<br>-Physical Check for Quantity and damage<br>- ଗୁଣବତ୍ତା ଏବଂ କ୍ଷୟକ୍ଷତି ପାଇଁ ବ୍ୟକ୍ତିଗତ ଭାବରେ ଯାଞ୍ଚ କରିବା		
		Status Tracking for Ordered supplies<br>ଅର୍ଡର ଦିଆଯାଇଥିବା ଯୋଗାଣଗୁଡ଼ିକ ପାଇଁ ସ୍ଥିତି ଟ୍ରାକ୍ କରିବା	**If the MIS is available for Supply Chain-**<br>**ଯଦି ଯୋଗାଣ ଶୃଙ୍ଖଳ ପାଇଁ ପରିଚାଳନା ବ୍ୟବସ୍ଥା (ଏମଆଇଏସ୍) ରହିଛି -**<br>Ability to check the delivery status (expected date)<br>ଡେଲିଭରି ସ୍ଥିତି ଯାଞ୍ଚ କରିବାର ସାମର୍ଥ୍ୟ (ଆଶା କରାଯାଉଥିବା ତାରିଖ)<br>**If the MIS is not available for supply chain-**<br>**ଯଦି ଯୋଗାଣ ଶୃଙ୍ଖଳ ପାଇଁ ଏମଆଇଏସ୍ ଉପଲବ୍ଧ ନଥାଏ** -<br>Able to communicate and ascertain the status of delivery<br>ଯୋଗାଯୋଗ କରିବା ପାଇଁ ଏବଂ ଡେଲିଭରି ସ୍ଥିତି ଧାର୍ଯ୍ୟ କରିବା ପାଇଁ ସକ୍ଷମ		
		Alternate Procurement Procedure<br>ଜିନିଷ ସଂଗ୍ରହ କରିବାର ବିକଳ୍ପ ପଦ୍ଧତି	**Local Purchase**<br>**ସ୍ଥାନୀୟ କ୍ରୟ**<br>Able to fill the Standard Formats<br>ମାନକ ଫର୍ମାଟଗୁଡ଼ିକ ପୂରଣ କରିବା ପାଇଁ ସକ୍ଷମ<br>Preservation of Purchasing documents<br>କ୍ରୟ ଦଲିଲଗୁଡ଼ିକ ସଂରକ୍ଷିତ		

			ଭାବରେ ରଖିବା<br>Any additional documentation required<br>ଅତିରିକ୍ତ କାଗଜପତ୍ର କାମ ଯଦି ଆବଶ୍ୟକ ହୁଏ<br>**Inter institutional Transfer**<br>**ଅନୁଷ୍ଠାନଗୁଡ଼ିକ ମଧ୍ୟରେ ସ୍ଥାନାନ୍ତରଣ**<br>Knowledge about the availability of required medical products in other institutions<br>ଅନ୍ୟ ଅନୁଷ୍ଠାନଗୁଡ଼ିକରେ ଆବଶ୍ୟକ ଚିକିତ୍ସାରେ ବ୍ୟବହୃତ ସାମଗ୍ରୀଗୁଡ଼ିକର ଉପଲବ୍ଧତା ବିଷୟରେ ଜ୍ଞାନ<br><br>Documentation for Medical Products transfer<br>ଚିକିତ୍ସାରେ ବ୍ୟବହୃତ ସାମଗ୍ରୀଗୁଡ଼ିକର ସ୍ଥାନାନ୍ତରଣ ପାଇଁ କାଗଜପତ୍ର କାମ କରିବା<br><br>Mechanism to transfer the medical products to your institutions<br>ଚିକିତ୍ସାରେ ବ୍ୟବହୃତ ସାମଗ୍ରୀଗୁଡ଼ିକ ଆପଣଙ୍କ ଅନୁଷ୍ଠାନକୁ ସ୍ଥାନାନ୍ତରଣ କରିବା ପାଇଁ ବ୍ୟବସ୍ଥା		
		SOP Development<br>କାର୍ଯ୍ୟ ଶୈଳୀ (ଏସ୍ଓପି) ପ୍ରସ୍ତୁତ କରିବା	-Identification of processes like, Stock Verification<br>- ପ୍ରକ୍ରିୟାଗୁଡ଼ିକର ଚିହ୍ନଟକରଣ ଯେପରିକି ଷ୍ଟକ ଯାଞ୍ଚକରଣ		
		Identification of Slow-moving medical products and corrective action to be required<br>ଅଳ୍ପ ବ୍ୟବହାର ହେଉଥିବା ଚିକିତ୍ସାରେ ବ୍ୟବହୃତ ସାମଗ୍ରୀଗୁଡ଼ିକର ଚିହ୍ନଟକରଣ ଏବଂ ଆବଶ୍ୟକ ହେଉଥିବା ସଂଶୋଧନାତ୍ମକ କାର୍ଯ୍ୟାନୁଷ୍ଠାନ	Verification of Stock register<br>ଷ୍ଟକ୍ ରେଜିଷ୍ଟରର ଯାଞ୍ଚକରଣ<br>Alerting the medical officer to inform about the slow-moving medical products to the higher centers.<br>ଅଳ୍ପ ବ୍ୟବହାର ହେଉଥିବା ଚିକିତ୍ସାରେ ବ୍ୟବହୃତ ସାମଗ୍ରୀଗୁଡ଼ିକ ବିଷୟରେ ମେଡିକାଲ୍ ଅଫିସର ବା ଡାକ୍ତରଙ୍କୁ ଜଣାଇବା, ଯେ କି ଉନ୍ନତ କେନ୍ଦ୍ରଗୁଡ଼ିକୁ ସୂଚନା ଦେବେ।		
		Preparation for attending any meeting<br>କୌଣସି ବୈଠକରେ ଯୋଗ ଦେବା ପାଇଁ ପ୍ରସ୍ତୁତ ହେବା	Understanding agenda<br>କାର୍ଯ୍ୟସୂଚୀ ବୁଝିବା<br><br>Based on Agenda, prepare the note for the points, needs to		

			express during the meeting<br>କାର୍ଯ୍ୟସୂଚୀ ଆଧାରରେ, ବିଭିନ୍ନ ବିଷୟ ପାଇଁ ବୈଠକରେ ପ୍ରକାଶ କରିବା ଆବଶ୍ୟକ ହେଉଥିବା ଟିପ୍ପଣୀ ପ୍ରସ୍ତୁତ କରନ୍ତୁ।		
		Risk Management Plan<br>ବିପଦ ପରିଚାଳନା ଯୋଜନା	In the event of Fire accident, what is your SOP?<br>ନିଆଁ ଲାଗିଗଲେ କାର୍ଯ୍ୟ ଶୈଳୀ (ଏସଓପି) ଅନୁଯାୟୀ ଆପଣ କ'ଣ କରିବେ?		
		Use of Microsoft Office Package- Word, Excel & Power point<br>ମାଇକ୍ରୋସଫ୍ଟ ଅଫିସ୍ ପ୍ୟାକେଜ୍ (Microsoft Office Package - ୱାର୍ଡ (Word) ଏକ୍ସେଲ୍ (Excel) ଏବଂ ପାୱାର ପଏଣ୍ଟ (Power point)ର ବ୍ୟବହାର	-Create file for Word, Excel & Power point<br>- ୱାର୍ଡ (Word) ଏକ୍ସେଲ୍ (Excel) ଏବଂ ପାୱାର ପଏଣ୍ଟ (Power point) ପାଇଁ ଫାଇଲ୍ ଉପୃନ୍ନ କରିବା<br><br>- Prepare a document in Microsoft Word, excel and Power point<br>- ୱାର୍ଡ, ଏକ୍ସେଲ୍ ଏବଂ ପାୱାର ପଏଣ୍ଟ ୱାର୍ଡ (Word) ଏକ୍ସେଲ୍ (Excel) ଏବଂ ପାୱାର ପଏଣ୍ଟ (Power point)ରେ ଏକ ଡକ୍ୟୁମେଣ୍ଟ ପ୍ରସ୍ତୁତ କରିବା		
		Stock Verification<br>ଷ୍ଟକ୍ ଯାଞ୍ଚକରଣ	Each item of pharmacy stock register needs to be verified physically through records of procurement, dispensing refund, indent/ issue, stock transfers, & stock taken last month.<br>ଫାର୍ମାସି ଷ୍ଟକ ରେଜିଷ୍ଟରର ପ୍ରତିଟି ଜିନିଷର କିଣା (procurement), ଫେରସ୍ତ, ବିତରଣ, ଇଣ୍ଡେଣ୍ଟ /ଜାରି, ଷ୍ଟକ୍ ଟ୍ରାନ୍ସଫର୍ ଏବଂ ଗତ ମାସରେ ନିଆଯାଇଥିବା ଷ୍ଟକ ତଥ୍ୟର ରେକର୍ଡ ବ୍ୟକ୍ତିଗତ ଭାବରେ ଯାଞ୍ଚ କରିବା।<br>The list for stock-taking has the following particulars:<br>ଷ୍ଟକ୍ ହିସାବ କରିବା ପାଇଁ ତାଲିକାରେ ନିମ୍ନଲିଖିତ ବିବରଣୀ ରହିଥାଏ:<br>*Name of the medicine, Quantity, Batch number, Expiry date, Manufacturing date, Manufacturer Name, Quantity counted, Quantity as per records, Quantity as per store, Discrepancy (if any), Discrepancy after reconciliation, Signature of the*		

			pharmacist;<br>*ଔଷଧର ନାମ, ପରିମାଣ, ବ୍ୟାଚ୍ ନମ୍ବର, ମିଆଦ ଶେଷ ତାରିଖ, ଉତ୍ପାଦନ ତାରିଖ, ଉତ୍ପାଦନକାରୀଙ୍କ ନାମ, ଗଣାଯାଇଥିବା ପରିମାଣ, ରେକର୍ଡ ଅନୁଯାୟୀ ପରିମାଣ, ଷ୍ଟୋର ଅନୁଯାୟୀ ପରିମାଣ, କୌଣସି ଅମେଳ, (ଯଦି କିଛି ଥାଏ), ସମନ୍ୱୟକରଣ ପରେ ଅମେଳ, ଫାର୍ମାସିଷ୍ଟଙ୍କର ସ୍ୱାକ୍ଷର;*		
3		Prescription Validation<br>ଡାକ୍ତରୀ ଚିଠାର ବୈଧତା ନିର୍ଦ୍ଧାରଣ	Facility Name<br>ବ୍ୟବସ୍ଥାର ନାମ<br>Medical Officer signature / initial<br>ଡାକ୍ତରଙ୍କର ସ୍ୱାକ୍ଷର/ ସଂକ୍ଷିପ୍ତ ସ୍ୱାକ୍ଷର		
		Reporting system for dispensing errors and near misses<br>ବିତରଣରେ ତ୍ରୁଟି ଏବଂ ତ୍ରୁଟି ପାଖାପାଖି ବିଷୟଗୁଡ଼ିକ ଜଣାଇବା ପାଇଁ ବ୍ୟବସ୍ଥା	Reporting Elements<br>ଜଣାଇବା ପାଇଁ ଉପାଦାନଗୁଡ଼ିକ<br>Name of the Reporter<br>ସୂଚନା ଦେଇଥିବା ବ୍ୟକ୍ତିଙ୍କର ନାମ		
		Monitoring Medication Adherence for Chronic diseases<br>ବହୁ ଦିନ ଧରି ଲାଗି ରହିଥିବା ରୋଗ ପାଇଁ ପରାମର୍ଶ ମୁତାବକ ଔଷଧ ସେବନ କରାଯାଉଛି ତତ୍ତ୍ୱାବଧାନ କରିବା ପାଇଁ	Pill Counting<br>ବଟିକା ଗଣିବା<br>Medication Refill Rate<br>ଔଷଧ ପୁନର୍ବାର ଭରିବା ହାର		
		Adverse Drug Reaction (ADR) - Events reporting<br>ଔଷଧ ଜନିତ ଅପ୍ରତ୍ୟାଶିତ ପ୍ରଭାବଗୁଡ଼ିକ (ଏଡିଆର୍) ଘଟଣାଗୁଡ଼ିକ ରିପୋର୍ଟ କରିବା ପାଇଁ ଫର୍ମ	Form Availability<br>ଫର୍ମ ଉପଲବ୍ଧତା<br><br>Fill form for Adverse drug reaction events<br>ଔଷଧଜନିତ ଅପ୍ରତ୍ୟାଶିତ ପ୍ରଭାବ ଘଟଣା ପାଇଁ ଫର୍ମ ପୂରଣ କରନ୍ତୁ		
4		Bio Medical Waste Management – SOP for Outreach Camps<br>ଚିକିତ୍ସାରୁ ଉତ୍ପନ୍ନ ବର୍ଜ୍ୟବସ୍ତୁ ପରିଚାଳନା - ଆଉଟରିଚ୍ କ୍ୟାମ୍ପ ବା ଶିବିର ପାଇଁ କାର୍ଯ୍ୟ ଶୈଳୀ (ଏସଓପି)	**Important Components** –<br>**ଗୁରୁତ୍ୱପୂର୍ଣ୍ଣ ଅଂଶଗୁଡ଼ିକ** –<br>Collection<br>ସଂଗ୍ରହ<br>Segregation<br>ପୃଥକୀକରଣ<br>Transportation<br>ପରିବହନ		
		Listing of emergency medicines during disaster management<br>ବିପର୍ଯ୍ୟୟର ପରିଚାଳନା	Listing of Common illness/ health conditions-Fever, Diarrhea etc..<br>ସାଧାରଣ ଅସୁସ୍ଥତା/ସ୍ୱାସ୍ଥ୍ୟ		

		ସମୟରେ ଜରୁରୀକାଳୀନ ଔଷଧଗୁଡ଼ିକର ତାଲିକା କରିବା	ଅବସ୍ଥାଗୁଡ଼ିକର ତାଲିକା କରିବା - ଜ୍ୱର, ଡାଇରିଆ ଇତ୍ୟାଦି□<br>Identification of medicines for the identified common illness/ health conditions<br>ଚିହ୍ନଟ କରାଯାଇଥିବା ସାଧାରଣ ଅସୁସ୍ଥତା/ସ୍ୱାସ୍ଥ୍ୟ ଅବସ୍ଥା ପାଇଁ ଔଷଧଗୁଡ଼ିକର ଚିହ୍ନଟକରଣ		
		First Aid<br>ପ୍ରାଥମିକ ଚିକିତ୍ସା	Condition-Wound management<br>ଅବସ୍ଥା -କ୍ଷତର ଚିକିତ୍ସା		
		Cardiopulmonary Resuscitation<br>କାର୍ଡିଓପଲମୋନାରୀ ରିସୋସିଟେସନ୍<br>(Resuscitation)	How to perform?<br>Steps-<br>କିପରି କରିବେ?<br>ପର୍ଯ୍ୟାୟଗୁଡ଼ିକ -<br><br>Check the area is safe<br>ଦେଖନ୍ତୁ ଯେ ଏହି କ୍ଷେତ୍ର ନିରାପଦ ରହିଛି<br>Shake and shout<br>ହାତ ହଲାଇ ବଡ଼ ପାଟିରେ ଡାକନ୍ତୁ<br>Call for hel<br>ସହାୟତା ପାଇଁ କଲ୍ କରନ୍ତୁ<br>Open airway by placing one hand on the forehead and the other 2 fingers under the chin<br>ଗୋଟିଏ ହାତ କପାଳରେ ଏବଂ ଅନ୍ୟ ଦୁଇ ଆଙ୍ଗୁଠି ଚିବୁକ ତଳେ ରଖି ଶ୍ୱାସ ମାର୍ଗ ଖୋଲିବାକୁ ଚେଷ୍ଟା କରନ୍ତୁ<br>Check for breathing for 10 seconds<br>ଶ୍ୱାସକ୍ରିୟା ପାଇଁ 10 ସେକେଣ୍ଡ ଯାଞ୍ଚ କରନ୍ତୁ<br>Clear chest & give 30 compressions with 2 hands, fingers and elbows locked, hands clasped<br>ଛାତି ଉପରେ କିଛି ରହିଥିଲେ ବାହାର କରି ଦିଅନ୍ତୁ ଏବଂ 2 ହାତରେ ଆଙ୍ଗୁଠି ଓ କହୁଣି ଛନ୍ଦି ହାତ ପରସ୍ପର ସହିତ ଛନ୍ଦି 30 ଥର ଚାପନ୍ତୁ<br>Close nose, open mouth, breathe into casualty's mouth, turn your head & breathe again ନାକ ବନ୍ଦ କରିଦିଅନ୍ତୁ, ପାଟି ଖୋଲନ୍ତୁ, ପୀଡ଼ିତଙ୍କର ପାଟି ଭିତରେ ନିଃଶ୍ୱାସ ଭରନ୍ତୁ, ଆପଣଙ୍କ ମୁଣ୍ଡ କଡ଼କୁ ନିଅନ୍ତୁ ଏବଂ ପୁଣି ଥରେ ନିଃଶ୍ୱାସ ଭରନ୍ତୁ		

			Repeat until help arrives<br>ସହାୟତା ପହଞ୍ଚିବା ପର୍ଯ୍ୟନ୍ତ ଏହା ଦୋହରାନ୍ତୁ		
10		Carry out basic tests/ measurements<br>ମୌଳିକ ପରୀକ୍ଷାଗୁଡ଼ିକ କରନ୍ତୁ /ମାପନ୍ତୁ	Blood Pressure<br>ଉଚ୍ଚ ରକ୍ତଚାପ<br>Hemoglobin<br>ହେମୋଗ୍ଲୋବିନ୍<br>BMI Calculation<br>ବିଏମଆଇ ହିସାବ କରିବା<br>Pregnancy Test<br>ଗର୍ଭାବସ୍ଥା ପରୀକ୍ଷା		
		Assemble/fit, use Oxygen cylinder, along with administration of oxygen<br>ଅକ୍ସିଜେନ୍ ସିଲିଣ୍ଡର୍ ଫିଟ୍ କରନ୍ତୁ, ଅକ୍ସିଜେନ୍ ଦେବା ସହ ବ୍ୟବହାର କରନ୍ତୁ			
		Nebulization<br>ନେବୁଲାଇଜେସନ୍			
		Wound Management<br>କ୍ଷତର ଚିକିତ୍ସା	Cleaning<br>ପରିଷ୍କାର କରିବା<br>Medicine Application or use Bandaging<br>ଔଷଧର ପ୍ରୟୋଗ କିମ୍ବା ବ୍ୟାଣ୍ଡେଜ୍‌ବ୍ୟବହାର କରିବା		
		Diagnosis- Malaria<br>ନିଦାନ -ମ୍ୟାଲେରିଆ	Symptoms<br>ଲକ୍ଷଣଗୁଡ଼ିକ<br>Signs<br>ସଙ୍କେତ<br>Rapid Diagnostic Tests<br>ରାପିଡ୍ ଡାଇଗ୍ନୋଷ୍ଟିକ୍ ଟେଷ୍ଟସ୍ (ଦ୍ରୁତ ନୈଦାନିକ ପରୀକ୍ଷାଗୁଡ଼ିକ)		
		Diagnosis- Upper Respiratory Tract Infection<br>ନିଦାନ - ଉପର ଶ୍ୱାସମାର୍ଗ ସଂକ୍ରମଣ	Symptoms<br>ଲକ୍ଷଣଗୁଡ଼ିକ<br>Signs<br>ସଙ୍କେତ		
		Diagnosis- Scabies<br>ନିଦାନ - ଯାଦୁ	Symptoms<br>ଲକ୍ଷଣଗୁଡ଼ିକ<br>Signs<br>ସଙ୍କେତ		

C.4. Simulation Exercise

C.4. ସିମୁଲେସନ୍ ଏକ୍ସରସାଇଜ୍ (ପରିସ୍ଥିତି ଅନୁଯାୟୀ ପଦକ୍ଷେପ ନେବା)

Domain Code<br>ଡୋମେନ ବା କ୍ଷେତ୍ର କୋଡ୍	Case<br>ପରିସ୍ଥିତି	Response (Satisfactory/ Unsatisfactory)<br>ଜବାବ<br>(ସନ୍ତୋଷଜନକ/ସନ୍ତୋଷଜନକ ନୁହେଁ)	Remarks<br>ଟିପ୍ପଣୀ
1	**Timely Corrective measures**<br>**ଠିକ୍ ସମୟରେ ସଂଶୋଧନାତ୍ମକ ପଦକ୍ଷେପଗୁଡ଼ିକ**<br><br>Assume that tomorrow you come to Pharmacy and engaged in your dispensing of medical products. At 11 :00 AM, you received the demand of Tetanus Toxoid from Medical Officer In charge for treating accident case. For which, you opened the refrigerator and found that refrigerator is out of order. In this condition, what would be your immediate plan of corrective action?<br>ଧରି ନିଅନ୍ତୁ ଯେ ଆସନ୍ତାକାଲି ଆପଣ ଫାର୍ମାସିକୁ ଆସନ୍ତି ଏବଂ ଆପଣଙ୍କ ଚିକିତ୍ସାରେ ବ୍ୟବହୃତ ସାମଗ୍ରୀଗୁଡ଼ିକର ବିତରଣରେ ନିୟୋଜିତ ହୁଅନ୍ତି□ ଦିନ 11ଟା ବେଳେ, ଏକ ଦୁର୍ଘଟଣା ରୋଗୀଙ୍କର ଚିକିତ୍ସା ପାଇଁ ମେଡିକାଲ୍ ଅଫିସର ଇନଚାର୍ଜଙ୍କଠାରୁ ଟିଟାନାସ ଟକ୍ସଏଡ (Tetanus Toxoid)ର ଆବଶ୍ୟକତା ପାଇଁ ଡିମାଣ୍ଡ ପାଆନ୍ତି□ ସେଥିପାଇଁ ଆପଣ ରେଫ୍ରିଜରେଟର୍ ଖୋଲନ୍ତି ଏବଂ ଦେଖନ୍ତି ଯେ ରେଫ୍ରିଜରେଟର ଚାଲୁ ନାହିଁ□ ଏହି ଅବସ୍ଥାରେ, ସଂଶୋଧନାତ୍ମକ କାର୍ଯ୍ୟାନୁଷ୍ଠାନ ପାଇଁ ଆପଣଙ୍କର ତତ୍କାଳ ଯୋଜନା କ'ଣ ହେବ?		
	**Reordering Time**<br>**ପୁନଃ ଅର୍ଡର ଦେବାର ସମୟ**<br><br>Suppose the monthly consumption of Gentamycin Injection is 50 vials. During summer season, you get to know that the possible serious bacterial infection in young infants has been rose more than three times. Considering this episode of sudden rise in cases, what are the parameters you will consider before making indenting to meet the demand of extra Gentamycin vials?<br>ଧରି ନିଆଯାଉ ଜେଣ୍ଟାମାଇସିନ୍ ଇଞ୍ଜେକ୍ସନ ମାସିକ 50 ଭାଏଲ୍ ଶେଷ ହୋଇଥାଏ□ ଖରା ଦିନେ ଆପଣ ଜାଣିବାକୁ ପାଆନ୍ତି ଯେ ଛୋଟ ପିଲାଙ୍କ କ୍ଷେତ୍ରରେ ବୀଜାଣୁଜନିତ ଗୁରୁତର ସଂକ୍ରମଣ 3 ଗୁଣରୁ ଅଧିକ ବୃଦ୍ଧି ପାଇଛି ଏହି ଘଟଣାଗୁଡ଼ିକରେ ଆକସ୍ମିକ ବୃଦ୍ଧିକୁ ବିଚାର କରି		Delivery Time and Quantity<br>ପହଞ୍ଚାଇବା ସମୟ ଏବଂ ପରିମାଣ

	ଆପଣ ଜେଣ୍ଟାମାଇସିନ୍ ଭାଏଲର ଅତିରିକ୍ତ ଆବଶ୍ୟକତା ପୂରଣ ପାଇଁ ଇଣ୍ଡେଣ୍ଟ କରିବା ପୂର୍ବରୁ କେଉଁ ମାନଦଣ୍ଡଗୁଡ଼ିକ ବିଚାର କରିବେ?		
	**Suggest Medical officer to alter medicine**<br>**ଔଷଧ ବଦଳାଇବା ପାଇଁ ମେଡିକାଲ୍ ଅଫିସରଙ୍କୁ ଅନୁରୋଧ କରନ୍ତୁ**<br><br>Assume that tomorrow you encounter stock out of Amoxycillin in your facility. In this case, which alternative medicine, you will prefer to suggest your medical officer?<br>ଧରି ନିଅନ୍ତୁ ଯେ ଆସନ୍ତାକାଲି ଆପଣଙ୍କ ବ୍ୟବସ୍ଥାରେ ଆମୋକ୍ସିସିଲିନ୍ ଷ୍ଟକ୍ ଶେଷ ହେବା ପରିସ୍ଥିତି ଉପୁଜେ□ ଏହିପରି ପରିସ୍ଥିତିରେ, ଆପଣ ଡାକ୍ତରଙ୍କୁ କେଉଁ ବିକଳ୍ପ ଔଷଧ ଦେବାକୁ କହିବାକୁ ଚାହିଁବେ?		Azithromycin<br>ଆଜିଥ୍ରୋମାଇସିନ୍
	**Possible Drug-Drug/Drug-Food Interaction:**<br>Iron and Calcium-<br>**ସମ୍ଭାବ୍ୟ ଔଷଧ ସହିତ ଔଷଧ / ଔଷଧ ସହିତ ଖାଦ୍ୟ ମଧ୍ୟରେ ପାରସ୍ପରିକ କ୍ରିୟା:**<br>ଲୌହସାର ଏବଂ କ୍ୟାଲସିୟମ୍<br><br>Assume tomorrow afternoon, a pregnant woman, comes to you with a prescription for Iron Folic Acid Tablet and Calcium Tablet. In the prescription, it mentions that both the tablets to be taken on daily basis. In this case, that is the possibility that the patient takes the medicine together. So, to avoid this situation of drug-drug interaction, what do you suggest?<br>ଧରି ନିଆଯାଉ ଆସନ୍ତାକାଲି ଅପରାହ୍ଣରେ ଜଣେ ଗର୍ଭବତୀ ମହିଳା ଆଇରନ୍ ଫୋଲିକ ଏସିଡ୍ ବଟିକା ଏବଂ କ୍ୟାଲସିୟମ୍ ବଟିକା ପାଇଁ ଡାକ୍ତରୀ ଚିଠା ସହିତ ଆପଣଙ୍କ ପାଖକୁ ଆସନ୍ତି□ ଏହି ଡାକ୍ତରୀ ଚିଠା ରେ, ଏହା ଦର୍ଶାଯାଇଛି ଯେ ଉଭୟ ବଟିକା ଦୈନିକ ଭିତ୍ତିରେ ସେବନ କରାଯିବ□ ଏହି ଘଟଣାରେ, ଏହା ସମ୍ଭବ ଯେ ରୋଗୀ ଉଭୟ ବଟିକା ଏକାଠି ସେବନ କରିଥାନ୍ତି□ ତେଣୁ, ଔଷଧ ସହିତ ଔଷଧର ପାରସ୍ପରିକ କ୍ରିୟା ପରିସ୍ଥିତି ଏଡ଼ାଇବା ପାଇଁ ଆପଣ କ'ଣ ପରାମର୍ଶ ଦେବେ?<br><br>Iron and Food-<br>ଲୌହସାର ଏବଂ ଖାଦ୍ୟ -<br><br>Suppose that a woman comes to you with		The time interval for taking these medicines should be at least 2 Hours. Through this interval, we may reduce the possible Drug-Drug interaction.<br>ଏହି ଔଷଧ ସେବନ କରିବା ମଧ୍ୟରେ ସମୟ ବ୍ୟବଧାନ ଅତିକମ୍‌ରେ 2 ଘଣ୍ଟା ରହିବା ଉଚିତ୍□ ଏହି ବ୍ୟବଧାନ ମାଧ୍ୟମରେ, ଆମେ ଔଷଧ ସହିତ ଔଷଧର ସମ୍ଭାବ୍ୟ ପାରସ୍ପରିକ କ୍ରିୟା ହ୍ରାସ କରିପାରିବା□<br><br>Taking Iron tablet after food will reduce iron absorption but it will promote adherence.<br>ଖାଦ୍ୟ ଖାଇବା ପରେ ଆଇରନ୍ ବଟିକା ଖାଇଲେ ଆଇରନ୍

	a complaint that she is feeling nauseating after taking Iron tablet. On enquiry, you find that the woman is taking Iron tablet in empty stomach. So, for this condition, what do you advise to the woman?<br>ଧରି ନିଆଯାଉ ଜଣେ ମହିଳା ଆପଣଙ୍କ ପାଖକୁ ଅଭିଯୋଗ ନେଇ ଆସନ୍ତି ଯେ ସେ ଆଇରନ୍ ବଟିକା ଖାଇବା ପରେ ବାନ୍ତି ମାଡ଼ିବା ପରି ଅନୁଭବ କରୁଛନ୍ତି□ ପଚାରି ବୁଝିଲା ପରେ ଆପଣ ଜାଣିବାକୁ ପାଆନ୍ତି ଯେ ମହିଳା ଜଣକ ଖାଲି ପେଟରେ ଆଇରନ୍ ବଟିକା ଖାଉଛନ୍ତି□ ତେଣୁ, ଏହି ଅବସ୍ଥା ପାଇଁ ଆପଣ ମହିଳାଙ୍କୁ କ'ଣ ପରାମର୍ଶ ଦେବେ?		ଅବଶୋଷଣ (absorption) ହ୍ରାସ ପାଇବ କିନ୍ତୁ ଏହା ନିୟମିତ ଔଷଧ ଖାଇବାକୁ ଉତ୍ସାହିତ କରିବ□

*************************************The End*************************************
