# Supplementary material for: Developing a competency assessment framework for pharmacists in primary health care settings in India
Source: PLoS One. 2025 Mar 10;20(3):e0316646. doi: 10.1371/journal.pone.0316646 (PMC11892806; doi:10.1371/journal.pone.0316646)
Supplement: Attachment — Submitted filename: Response to Reviewers.docx [file pone.0316646.s004.docx]

**Rebuttal Letter**

For the Manuscript- Developing a competency assessment framework for pharmacists in primary health care settings in India

PONE-D-24-33395

Date: 2nd Nov 2024

| **Sr. No.** | **Section** | **Comments** | **Response** | **Description-Response (Page and Line Number** |
| --- | --- | --- | --- | --- |
| 1 | Abstract | In the background, it's crucial to highlight the significance of the competency framework for pharmacists, as it underpins their professional development and performance. | Referring to the suggestion, I made the required changes. | Please refer to ln No. 35 to 40 on page 2 |
| 2 |  | The method of assessment was insufficiently described. | Referring to the suggestion, I made the required changes. | Please refer to ln No. 42 to 49 on page 2 |
| 3 |  | To whom were the competency framework and associated behaviour discussed and agreed upon? Please clarify the statement. | Referring to the suggestion, I made the required changes. | Please refer to ln No. 53 to 54 on page 2 |
| 4 |  | In general, there is a lack of information about the competencies, just the number of competencies and the number of roles. Kindly add some lines in the result or conclusion about which competency framework for pharmacists has been introduced. | Referring to the suggestion, I made the required changes. | Please refer to ln No. 88 to 101 on page 3. |
| 5 | Introduction | Please add an example from the primary rather than the community set-up to elaborate on the importance of competency at the primary healthcare level. | I tried my best to include articles from primary healthcare settings. |  |
| 6 |  | Add a few lines about the availability of pharmacists at the primary healthcare level and then explain their importance to their country's prospects. | Referring the suggestion, made the necessary changes. | Please refer to ln No. 198 to 202 on page 6. |
| 7 |  | The authors are advised to specify the status of pharmacists at their country level because these studies are of Indian origin. So, the targeted approach will be more important than covering the whole world. | Yes, I tried my best to share status in an Indian setting | Please refer to ln No. 211 to 217 on page 6. |
| 8 |  | Please advise the authors to reference the statements, as no competency assessment has already been conducted in their country | We learned from literature search and expert consultation that there is no competency assessment framework. |  |
| 9 |  | There is no introduction regarding the competency tool and globally available competencies for pharmacists at the primary level. | We discussed about the FIP competency framework and later in stage 1, we listed the competencies under different domains from various studies. | Please refer to ln No. 179 to 197 on pages 5 and 6. |
| 10 | Method | Authors are advised to use the assessment tool for literature review for effective data collection and representation. | In stage 1, we used a systematic approach to searching literature through a defined search strategy and followed PRISMA guidelines. Please refer to Fig 1 for details. |  |
| 11 |  | In stage two, on which basis the experts were selected for consultation? | We identified experts based on selection criteria such as education, years of experience, and current role. Please refer to the experts section. | Please refer to ln No. 277 to 281 on page 8. |
| 12 |  | In stage three, which instrument was used for assessing competencies? Please specify. | Referring to the suggestion, we made the changes. | Please refer to ln No. 268 to 269 on page 8. |
| 13 |  | There should be numbered score definitions in the assessment tool. | We developed the score definition for the instruments assessing skills as Observational checklists and mini-clinical exercises. Please refer to table 8 Score definition. | Please refer to table 5 on page 14 |
| 14 |  | Is there only English grammar pretesting required for the competency assessment tool? | No, we pre-tested the competency assessment tool in bilingual format in English and Odia. | Please refer to ln no 466 and 467. |
| **15** | Results | The competencies selected in the literature review are related to the in-service pharmacist or not. These competencies were related to pharmacists or taken as general. | Yes, the literature review is related to the pharmacists working in primary health care settings. |  |
| **16** |  | On which basis were the eleven roles made for pharmacist competencies, and on which bases were they selected? | In stage 2, during the consultation, it emerged that rather than considering the domain, it is better to use the role as a basis to define competencies. These roles are primarily based on the Indian Public Health Standard, 2022, and later on, the consensus among experts, 11 roles were finalized. | Please refer to ln no 318-320 on page 9. |
| **17** |  | In role 2, how is it possible for a pharmacist to observe the optimal use of medical products? | In role 2, one of the competencies is- Able to encourage the use of medical products.  The pharmacist can encourage patients to return unused, unwanted, or expired medicines to the Pharmacy for safe disposal. Pharmacists can also maintain records of returned medical products to monitor usage further.  The additional file mentions these two ways as behaviors for the said competencies. For details, please refer to Additional file 2. | Please refer to additional file 2. |
| **18** |  | In role 3, please specify which drugs require high compliance with regulations. | Medical products with narcotics and psychotropic agents require high compliance with regulations.  We made the suggested changes. | Please refer to ln no 362-363 on page 12. |
| **19** |  | In role 4, although medical product management and documentation are solely the responsibility of the pharmacist as they are available in the pharmacy, the pharmacist assists the medical officer with these tasks. | Primary health Centres conduct various activities for public health programs, including Leprosy, Tuberculosis, and Vector disease control programs. In those cases, the Pharmacist is supposed to assist the medical officer with medical product management and documentation. |  |
| **20** |  | In role 7, on which limit the ethical practice will be allowed. Please specified. | Regarding ethical practice, the consensus made among the experts as one of the behaviors is -  “Be well versed with the Code of Ethics of the Pharmacy Council of India and as given in Pharmacy Practice Regulation.” (Please refer to additional file 2 )  Made the required changes in the manuscript. | Please refer to additional file 2. |
| **21** |  | In role 10, Is it possible for the author to please specify the list of diagnostic tests and checks in emergencies? | We specified the list of diagnostics in the manuscript. These tests are described as behaviors in Additional File 2. | Please refer to additional file 2 |
| **22** |  | In role 11, authors are advised to please specify the programs for continuing professional development in Indian origin. | In India, there is no structured program for pharmacists' continuing professional development. The National Health Mission, along with other initiatives, organizes capacity-building programs regularly. In light of this, one behavior that emerged from expert consensus is actively participating in the various training programs offered by the National Health Mission and other health programs established by the Ministry of Health and Family Welfare (MoHFW) and State Health Departments.  For details, please refer to additional file 2. | Please refer to additional file 2. |
| **23** |  | Authors are advised to reduce the paragraph content as already described in the table. | We removed two tables-table 4 and 5. |  |
| **24** |  | Authors are advised to provide the details of the validation of the competency assessment tool. Which tests were performed during the pre-testing of the competency assessment tool? | We developed the CAT using a content validation (Stage 4) approach. Later, under stage 5, we pre-tested the CAT in one of the public health facilities to understand the construct regarding language clarity and local programmatic variations. | Please refer to ln no 467 to 475 on pages 15 and 16. |
| **25** | Discussion | The discussion regarding the factors related to coping with pre-service education is limited. The authors advised adding more possible measures to cope with these challenges at the government level. | We made the needful changes. | Please refer to ln no 493 to 515 on pages 16 and 17. |
| **26** |  | The discussion regarding the importance and implications of competence assessment was very limited, making this portion weak. | We made the needful changes. | Please refer to ln no 522 to 536 on page 17. |
